# The demands–control–support work stress model and risk of ischemic heart disease: causal inference based on observational epidemiology

**DOI:** 10.5271/sjweh.4299

**Published:** 2026-07-01

**Authors:** Jens Peter Bonde, Stinna Skaaby, Esben M Flachs, Maureen Dollard, Katherine Keyes, Annika Rosengren, Ingrid AS Mehlum, Sigurd Mikkelsen

**Affiliations:** 1 Department of Occupational and Environmental Medicine, Bispebjerg and Frederiksberg University Hospital, Copenhagen, Denmark.; 2 Institute of Public Health, University of Copenhagen, Copenhagen, Denmark.; 3 Psychosocial Safety Climate Global Observatory, University of South Australia, Adelaide, Australia.; 4 Department of Epidemiology, Columbia University, New York, USA.; 5 Institute of Medicine, Sahlgrenska Academy, University of Gothenburg, Gothenburg, Sweden.

**Keywords:** cardiology, occupational exposure, psychosocial exposure, risk estimate, workplace

## Abstract

**Objective:**

Reviews consistently suggest an association between job strain and ischemic heart disease (IHD), but causality remains uncertain. This study aimed to critically assess causal inference using the most informative epidemiological studies.

**Methods:**

A systematic search in Pubmed and Embase up to 15 November 2024 identified observational studies reporting quantitative estimates of associations between job strain (defined by job demands and control) and IHD. Eligible studies were cohort or case–control designs with exposure data obtained independently by medically verified IHD (ICD-8/9: 410–414; ICD-10: I20–I25) and risk estimates adjusted at least for age, sex, and socio-economic status. One estimate per study was included in inverse-variance weighted random-effects meta-analyses. We evaluated main sources of upward and downward bias, potential confounding, and key criteria for causal inference including outcome specificity, exposure–response, and consistency.

**Results:**

This review comprised 25 cohort and 1 case–control study (122 risk estimates). The fully adjusted pooled relative risk estimate (RRE) for job strain and all IHD outcomes combined was 1.14 [95% confidence interval (CI) 1.06–1.23; 21 studies]. For myocardial infarction, the RRE was 1.08 (95% CI 1.00–1.15; 11 studies), and, in studies using job-exposure matrices, it was 1.06 (95% CI 0.99–1.13; 7 studies). Strong heterogeneity, small effect sizes, limited exposure–response evidence, net bias in unpredictable directions, and lack of confirmation of findings in studies using alternatives to self-reported exposure assessment preclude causal inference.

**Conclusion:**

Evidence for a causal relationship between job strain and IHD is limited. At most, any true effect appears to be small.

The risk of ischemic heart disease (IHD) associated with work-related psychosocial exposures has increasingly been addressed by occupational epidemiology over the past 50 years. This sustained interest is evident in numerous reviews (1–8) and two meta-reviews (9, 10). Among a range of different types of exposures, job strain – defined by Karasek's job content (JC) model of work stress introduced in the late 1970s (11) – is the most extensively studied. The model proposes that high psychological demands (ie, workload, time pressure) combined with low job control (ie, limited decision authority and skill discretion) is hazardous to health. Subsequently, low social support from supervisors and colleagues has been suggested to amplify adverse health effects of job strain, labelled iso-strain (iso referring to isolation) (12).

Several reviews have consistently reported associations between job strain and IHD risk. Yet, despite decades of research, it remains controversial if associations are causal (13–18). Most studies rely on self-reported assessment of psychosocial demands and job control through questionnaires, which may introduce bias and artificially inflate associations (19). Self-reports may also vary across time, populations and social groups and thus compromise comparability within and between studies (14). Moreover, determinants of IHD such as early life adversity and psychiatric morbidity may also be related to job strain and are rarely accounted for, leaving the possibility of residual confounding (15, 17, 18, 20). Uncertainty also persists regarding exposure–response relationships and the role of the core components of the JC model. Specifically, it remains unclear if IHD associations with a combination of high demands and low control are driven by their independent effects or if there is also an interaction effect and what the relative effect sizes of these three components are.

With an explicit focus on job strain, this review enables a more detailed and critical evaluation of causal inference, something which broader reviews covering multiple psychosocial exposures cannot easily accommodate. Although only a limited number of new studies have been published since the most recent comprehensive review (8), the narrow scope of the present review allows for closer scrutiny of methodological issues and an explicit focus on aspects relevant to causal inference, such as consistency, temporality, strength of associations, exposure–response patterns, and the specificity of exposure and outcome.

Given that reliance on self-reported exposure is considered a major limitation, we examine whether findings are supported by studies employing alternative measures of exposure such as survey-based and expert-rated job-exposure matrices (JEM). We deliberately avoid generic study quality assessment tools, which have been criticized for their subjectivity and lack of transparency (21, 22). Instead, the intention was to identify and highlight key methodological challenges inherent in this particular field of research. These include outcome specificity, exposure–response patterns, bias inflating or attenuating risk estimates, confounding and effect modification by socio-economic position, and evidence derived from studies with independent or objective exposure data.

In summary, the aim of this review, accordingly, was to systematically and critically assess the evidence that the association between job strain and IHD is causal.

## Methods

### Literature search and data extraction

We performed systematic literature searches in Pubmed and Embase in mid-November 2024. The search strategy combined MeSH terms and free-text keywords related to study design, exposure, and outcome (see the supplementary material, URL, I). Inclusion and exclusion criteria are outlined in table 1. The journal paper selection process from the initial yield of 3396 records to the final 26 eligible peer-reviewed original studies is shown in supplementary material II. To ensure complete coverage, we compared our final set of included studies with those reported in previous systematic reviews (4, 6–8, 13, 23). Detailed characteristics of the included papers and reasons for excluding full-text articles are provided in supplementary materials III and IV and an overview of type of exposures and covariates included in analyses in supplementary materials V and VI.

**Table 1:** Eligibility of original journal articles. [IHD=ischemic heart disease.]

**Inclusion criteria**	
Study content	Original peer-reviewed journal papers in English providing quantitative estimates of associations between job strain and risk of IHD.
Study design	Cohort and nested case–control studies with outcome data collected independently of exposure data. Hospital-based case–control and cross-sectional studies if exposure data were collected independently of outcomes (eg, studies using objective exposure metrics).
Outcome	IHD (ICD-8-9: 410-414; ICD-10: I20-I25, including angina pectoris but not hypertension) ascertained by hospital discharge diagnoses, death certificates or clinical examinations.
Analysis	Risk estimates minimally adjusted for sex, age, and socioeconomic status (eg, income, education, occupation, or employment grade) through analysis, stratification, or study design.
**Exclusion criteria**	
Exposure	Exposure data based on individual recall after occurrence of the outcome.
Outcome	Fully or partially self-reported outcomes, including self-reported physician diagnoses.
Other	Studies with overlapping populations and analyses. Only the most recent or most informative study was included.
	Studies with selective reporting of results in relation to the stated objectives.

We extracted data on demographic characteristics, study population, design, exposure and outcome definitions, statistical analysis, and adjustment for confounders using predefined templates. Fully adjusted risk estimates were collected for all reported exposure levels, IHD outcomes (overall IHD, myocardial infarction, unspecified IHD, and IHD mortality), and relevant subgroups. These fully adjusted risk estimates were, in addition to sex, age and some measure of socioeconomic class (cf. table 1) adjusted for the most complete range of control variables for each particular study. Thus, the level of full adjustment differs across studies. Where available, the least adjusted estimates (typically only adjusted for age and sex) were also recorded to evaluate the impact of additional covariate adjustments.

To ensure consistency, risk estimates were transformed so that they always reflected the effect of supposed harmful exposures. Thus, while high psychological demands were expected to increase risk, protective factors such as high job control or high workplace social support were inverted to align with this approach. Because transformation of confidence intervals was not readily possible for intermediate exposure levels, these were excluded from the meta-analyses, but original data were kept in forest plots and paper summaries (supplementary material, III). Given the relative rarity of IHD in the age-range of gainfully employed people (24), hazard ratios (HR), relative risks, and odds ratios were treated as equivalent measures of associations and labelled relative risk estimate (RRE) in the following unless otherwise specified.

At least two authors independently screened titles, abstracts, and full-text papers, resolving discrepancies through discussion. One author performed data extraction, which two others independently verififed.

### Data synthesis and statistical analysis

We summarized risk estimates using forest plots. When multiple estimates were reported for the same exposure–outcome relationship for various subgroups within a study (eg, sex or occupational grade), we calculated a fixed-effect, inverse-variance-weighted average using the metafor package (REML option) in R (R Foundation for Statistical Computing, Vienna, Austria). However, if risk was reported by several exposure levels, only the highest level was included in pooled analyses, ensuring that each study contributed only one estimate per exposure–outcome combination. One study reported risk estimates for two different IHD outcomes, myocardial infarction and revascularization (25).

Across studies, we applied random-effects models regardless of between-study heterogeneity due to variations in populations, exposure definitions, and measurement methods. Publication bias was assessed visually using funnel plots, with asymmetry suggesting possible bias.

Several articles from the IPD-Work Consortium included risk estimates from cohorts in various European countries that have not been published at the time of this writing. For these, we calculated a weighted random-effects estimate with 95% confidence intervals (CI), excluding cohorts already published separately [eg (26), excluded from the summary estimate in (27)] and those that did not fulfil our inclusion criteria.

Primary analyses assessed the overall risk of IHD by combining myocardial infarction, unspecified IHD (including angina pectoris and other clinical manifestations of IHD), and IHD mortality. Supplementary analyses explored whether associations varied by outcome definition, sex, socioeconomic position, exposure assessment method, and inclusion of potential effect modifiers (eg, behavioral and medical risk factors) in statistical models.

The review protocol was registered with the International Prospective Register of Systematic Reviews (PROSPERO: CRD42025617675) and follows the PRISMA guidelines for systematic reviews (28).

## Results

### Characteristics of included studies

A total of 25 prospective cohort studies and 1 case–control study (29) were eligible according to our inclusion criteria. These studies were published between 1994 and 2023 and contributed 122 risk estimates (table 2); of these, 11 (14 risk estimates) examined the association between job strain and the most specific of the three outcomes (myocardial infarction).

**Table 2:** Characteristics of follow-up studies reported in 26 publications 1994–2023 addressing risk of ischemic heart disease (IHD) according to the demands-control-support job stress model. [JEM=job-exposure matrix]

		Studies by outcome (N)			
		Total	Myocardial infarction ^1^	IHD unspecified ^2^	IHD mortality
N (all)		26 ^3^	7	17	3
Countries					
	Nordic countries	10	3	6	1
	UK	1	1	0	0
	Europe, other or several countries	5	1	3	1
	USA and Canada	10	2	8	1
Study populations					
	Population-based, national samples	6	2	3	1
	Populations-based, other samples	8	1	8	0
	Occupational samples	6	1	3	2
	White-collar workers	4	2	2	0
	Blue-collar workers	1	0	1	0
	Other (case–control studies)	1	1	0	0
Exposure ascertainment					
	Self-administered questionnaire or interview	16	5	12	0
	JEM, survey based	7	2	3	2
	JEM, expert based	2	0	1	1
	Company rosters, registers, managers, work units	1	0	1	0
Outcome ascertainment					
	Hospital diagnosis or death register	17	5	9	3
	Medically verified self-report	5	2	4	0
	Clinical examination	3	0	3	0
	Other	1	0	1	0
Sex distribution					
	0–25% women	10	3	5	2
	>25–50% women	11	3	7	1
	>50% women	5	1	5	0
	Only men	8	1	5	2
	Only women	4	1	4	0
	Both men and women	14	5	8	1
Primary participation of eligible					
	<75%	6	4	5	0
	5– <100%	7	2	4	1
	100%	6	1	4	1
	Missing	7	0	4	1
Follow-up, entry year					
	Before 1990	13	4	6	3
	1990– <2000	8	2	7	0
	≥2000	5	1	4	0
Follow-up, duration, years ^4^					
	1–5	3	0	3	0
	6–10	3	0	3	0
	>10	17	5	10	3
	Missing	3	2	1	0
Number of cases					
	<100	1	0	1	0
	100– <500	11	3	7	1
	500– <1000	6	2	5	0
	≥1000	8	2	4	2

^1^ Myocardial infarction: ICD-8-9: 410; ICD-10: I21. ^2^ IHD: ICD-8-9: 410-414; ICD-10: I20-I25. ^3^ Studies do not sum to 26 as one study may contribute to several outcomes. ^4^ Not including case–control studies.

In most cohort studies, exposure to psychosocial factors was self-reported at baseline (table 2). Most of these used abbreviated or adapted versions of the original Job Content Questionnaire (JCQ) (30, 31), often with modified items or response options (32). Where reported, internal consistency (eg, Cronbach’s alpha) for the subscales on demands, control, and social support was typically >0.65, though exceptions existed (33, 34). Factor analyses, such as one conducted in a large French population, largely confirmed the JCQ’s dimensional structure (30).

Ten studies used JEM or other alternatives to individual self-reported exposure assessment (table 2). JEM were developed from population surveys in seven studies (35–41) and based upon expert evaluations or work unit data in three studies (42–44). Exposure was usually assessed only at baseline, but three studies included repeated measures (36, 38, 45); two of these examined cumulative exposure over time (38, 45).

IHD outcomes were generally ascertained through hospital discharge records or death certificates, although a few relied on clinical examinations during follow-up (table 2).

Most studies adjusted for behavioral and clinical risk factors for IHD. However, few accounted for other potentially important confounders such as family history of cardiovascular disease, childhood adversity, or social network (see supplementary material VI).

In all but one study, job strain was dichotomized based on the median values of demands and control scores. The high-strain group was compared either with the low-strain (or “relaxed”) group (17 studies), all other quadrants combined (27, 35, 46), or alternative reference groups (47).

The separate independent effect of job demands, job control, and social support were investigated in 16, 20, and 8 studies, respectively. In 14 studies, the level of one or more of these exposures measured on continuous scales were divided into tertiles or quartiles, allowing for exposure–response analyses. On the contrary, only one study assessed exposure–response for job strain (39).

Studies based on self-reported measures classified exposure levels exclusively according to quantiles of questionnaire score distributions. This is a convenient approach within individual studies. However, these cut-points are population-specific but rarely reported, and therefore obscure the absolute level of exposure and make comparisons of exposure intensity across studies difficult. Moreover, the composition of absolute levels by quantile categories in specific studies is also obscured. Thus, commonly used upper quantiles may include large proportions of participants with low absolute exposure levels. Iso-strain, defined as high strain combined with low social support, was examined in two studies, yielding three risk estimates.

### Risk estimates

**Figure 1:**
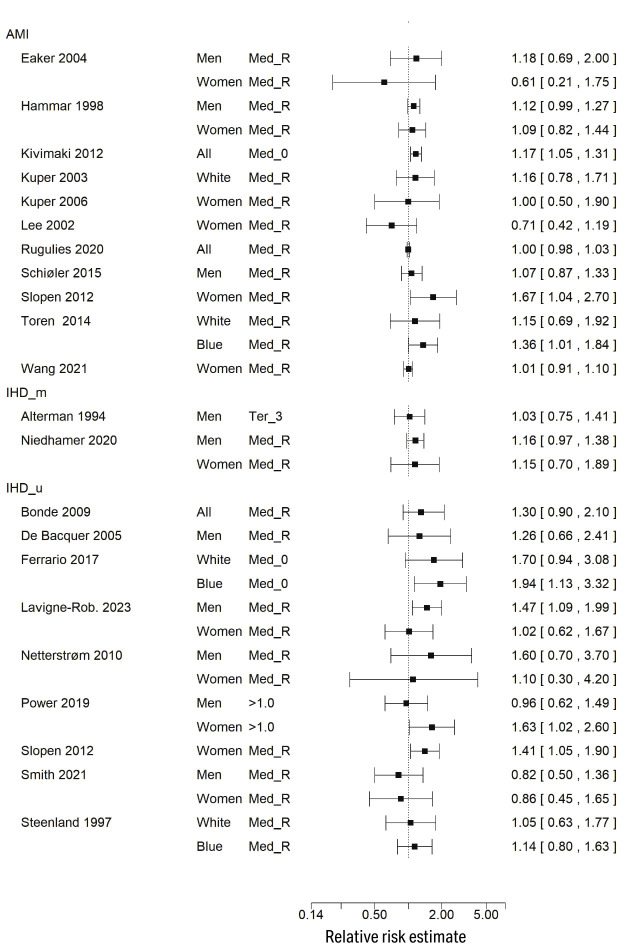
Forest plot of relative risk estimate (RRE) of ischemic heart disease (IHD) by high versus low jobstrain in eligible published studies (N=21 studies with 22 estimates). RRE refer to approximative relative risks in studies with rare outcomes (true relative risks, hazard ratios and odds ratios). The meta-analytic weighted risk estimates across studies are not provided given strong heterogeneity across studies. [AMI=acute myocardial infarction; IHD_m=IHD mortality; IHD_u= IHD unspecified; white_c= white-collar worker; blue_c= blue-collar worker; Med_R=high versus low job strain defined by median scale score, quadrant method, strain versus relaxed; Med_O=high versus low job strain defined by median scale score, quadrant method, strain versus all others; > 1.0= the ratio of demands and inversed control scores ≥1 versus <1.0.]

*Job strain.* The variance-weighted random-effects estimate across all 21 studies addressing this exposure regardless of how it was measured indicated an elevated risk of IHD associated with job strain (figure 1). The association was strongest for unspecified IHD and weaker for myocardial infarction and IHD mortality (table 3). Risk estimates in individual studies ranged from RRE 0.61–1.94. Of the 32 included estimates, 22% (N=7) were <1.00, 47% (N=15) were 1.01–1.25, and 31% (N=10) were >1.25. Overall, 22% of estimates were statistically significant. There was some indication of publication bias (supplementary material VII).

**Table 3:** Meta-analytic fully adjusted random effect risk estimates (RRE) ^1^ with 95% confidence interval (CI) for associations between demands–control–support variables and ischemic heart disease (IHD) (N=26 studies). RRE were based on the fully adjusted risk estimates of each study, minimally adjusted for effects of sex, age and some measure of socio-economic position, and additionally for a varying number of other potential confounders, primarily health behaviour (smoking, alcohol consumption, leisure time physical activity) and medical factors (hypertension, diabetes, hyperlipidaemia). Within-study stratified risk estimates were pooled, so each study contributes one risk estimate to the meta-analytic across-study analyses. If risk was provided by exposure level, only the highest level was used.

Exposure		Studies (N)	Estimates (N)	RRE	95% CI	I ^2%^ (P-value)
High job demands						
	IHD overall	16	24	1.03	0.96–1.11	71 (0.40)
	Myocardial infarction	7	10	1.06	0.94–1.18	80 (0.36)
	IHD unspecified	6	10	1.00	0.97–1.03	.
	IHD mortality	3	4	1.01	0.87–1.18	62 (0.89)
Low job control						
	IHD overall	20	30	1.10	1.03–1.18	56 (0.01)
	Myocardial infarction	9	13	1.08	1.02–1.14	21 (0.01)
	IHD unspecified	8	13	1.02	0.88–1.18	46 (0.79)
	IHD mortality	3	4	1.12	1.05–1.19	
Low social support						
	IHD overall	8	11	1.10	0.99–1.24	26 (0.09)
	Myocardial infarction	3	3	1.06	0.94–1.19	6 (0.36)
	IHD unspecified	4	6	1.25	0.85–1.85	72 (0.26)
	IHD mortality	1	2	1.21	0.99–1.48	
High job strain						
	IHD overall	21 ^2^	32	1.14	1.06–1.23	60 (0.00)
	Myocardial infarction	11	14	1.08	1.00–1.15	49 (0.04)
	IHD unspecified	9	15	1.26	1.09–1.47	18 (0.00)
	IHD mortality	2	3	1.13	0.98–1.31	
High isostrain						
	IHD overall	2	3	1.46	1.12–1.91	
	Myocardial infarction	0	0			
	IHD unspecified	1	1	1.92	1.05–3.52	
	IHD mortality	1	2	1.14	0.96–1.35	

^1^ RRE refers to approximative relative risk in studies with rare outcomes (true relative risk, hazard ratio and odds ratio). ^2^ The number of studies does not add up to the total number (N=21), because one study (Slopen et al, 2012) contributed with two estimates (myocardial infarction and IHD unspecified).

Exploratory subgroup analyses suggested higher risks in studies conducted outside the Nordic countries, in occupational samples, among men and blue-collar workers, and in studies using self-reported exposure data. Elevated risks were also more frequently observed in studies with lower response rates, smaller sample sizes, non-registry-based diagnoses, and longer follow-up durations (table 4). On average, adjusting for behavioral and medical risk factors attenuated risk estimates. However, differences between subgroups were small and confidence intervals largely overlapped.

**Table 4:** Meta-analytic fully adjusted random effect risk estimates (RRE)^1^ with 95% confidence interval (CI) for associations between job strain and ischemic heart disease by study characteristics (N=21 studies). RRE were based on the fully adjusted risk estimates of each study, minimally adjusted for effects of sex, age and some measure of socio-economic position, and additionally for a varying number of other potential confounders, primarily health behaviour (smoking, alcohol consumption, leisure time physical activity) and medical factors (hypertension, diabetes, hyperlipidaemia). Within-study stratified risk estimates were pooled, so each study contributes one risk estimate to the meta-analytic across-study analyses. If risk was provided by exposure level, only the highest level was used.

Characteristic		Studies (N)	Estimates (N)	RRE	95% CI	I ^2%^ (P-value)
Country						
	Nordic	7	10	1.09	0.99–1.19	45 (0.09)
	Other	14	22	1.14	1.04–1.25	39 (0.01)
Population						
	Occupational	10	13	1.16	1.08–1.25	
	Other	11	19	1.09	1.00–1.20	60 (0.06)
Exposure ascertainment						
	Self-report	13	20	1.18	1.06–1.32	27 (0.01)
	Job-exposure matrix	7	11	1.06	0.99–1.13	47 (0.08)
	Other	1	1	1.30	0.85–1.99	
Outcome ascertainment						
	Hospital diagnosis or death registry	13	20	1.11	1.04–1.19	45 (0.00)
	Other	8	12	1.19	1.02–1.40	57 (0.03)
Baseline response rate						
	<75%	7	12	1.38	1.19–1.60	
	75–100%	7	8	1.09	0.99–1.20	51 (0.07)
	Missing	7	12			
Entry year						
	1970–1989	10	16	1.15	1.08–1.22	
	≥1990	11	16	1.09	0.97–1.24	65 (0.16)
Follow-up duration, years						
	1–10	6	7	1.08	0.95–1.22	55 (0.24)
	>10	13	21	1.16	1.05–1.28	42 (0.01)
	Missing	2	4			
Sample size						
	<500	9	12	1.17	0.97–1.41	27 (0.11)
	≥500	12	20	1.11	1.03–1.19	59 (0.01)
Studies with potential mediators ^2^						
	Yes, adjusted	14	20	1.12	1.03-1.23	31 (0,01)
	Yes, not adjusted	14	20	1.24	1.14-1.35	22 (0,00)
	No	7	12	1.12	1.01–1.25	61 (0.04)
Sex strata ^3^						
	Men	13	16	1.16	1.08–1.25	
	Women	11	12	1.10	0.94–1.28	38 (0.23)
	Unspecified	4	4	1.09	0.96–1.24	66 (0.17)
Social strata ^3^						
	White collar	8	10	1.25	1.08–1.45	9 (0.00)
	Blue collar	4	4	1.24	1.02–1.50	32 (0.03)
	Unspecified	12	18	1.06	1.00–1.13	41 (0.05)
Job strain adjusted for demands and control						
	Yes ^4^ without adjustment	4	5	1.14	0.94–1.38	61 (0,18)
	Yes ^4^ with adjustment	4	5	1.37	1.11–1.70	57 (0,00)
	No ^5^	17	27	1.12	1.04–1.20	41 (0.00)

^1^ RRE (relative risk estimate) refers to approximative relative risk in studies with rare outcomes (true relative risk, hazard ratio and odds ratio). ^2^ Only studies with and without adjustment for behavioural and/or medical CVD risk factors. ^3^ The number of studies does not add to total number = 21 because several studies provide risk estimates for both men and women and/or both white and blue collars. ^4^ The only four studies that provide risk estimates with and without adjustment for effects of demands and control (to allow assessment of this adjustment). ^5^ Studies with no adjustment for effects of demands and control.

One large Swedish case–control study (29) used a JEM based upon a population survey of self-reports to assess exposure. The findings were close to the average of the cohort studies (figure 1).

Three studies examined the interaction between high demands and low control using a multiplicative model (26, 43, 48); none reported a significant interaction effect. Studies using the quadrant method generally did not adjust effects of job strain for main effects of demands and control.

In seven studies that used survey-based or expert-rated JEM, the average excess risk of IHD was about one-third of the excess risk found in studies using self-reported exposures (table 4). However, results from these studies were heterogeneous. One study found increased risk of myocardial infarction among male blue-collar but not white-collar workers (37). Another found short- and long-term effects in an occupational sample, which disappeared after adjusting for education (45). Five other studies did not report significant associations between job strain and IHD (29, 38, 40, 43, 48).

With two notable exceptions, no studies evaluated effects of cumulative exposure or timing of exposure relative to the outcome. First, a French mortality study assessed job strain in each of the years from 1976–2002 using a survey-based JEM (38). Cumulative job strain across all years was associated with IHD among men (HR 1.24, 95% CI 1.04–1.48) but not women (HR 1.04, 95% CI 0.65–1.67). Similar effects were observed for current exposure and a measure of cumulative exposure giving more weight to recent exposure. However, effects were not adjusted by job demands or decision latitude, and in particular the latter may have confounded the effect of job strain. Adjustment for socio-economic class was by various JEM-derived chemical and physical exposures rather than by income or education. Second, a Danish cohort study addressed risk of fatal and non-fatal myocardial infarction in a large occupational sample. Exposures were derived from a sex-, age- and calendar-year-specific JEM (39). Job strain at baseline was associated with increased risk over the follow-up period. The risk was higher when job strain was defined by quartiles rather than medians (adjusted HR 1.24, 95% CI 1.19–1.29 versus 1.07, 95% CI 1.03–1.10). These models adjusted for income, but – when education was added – the baseline association disappeared. No consistent increase in risk was observed with a higher number of exposed years, if anything, the opposite.

*Iso-strain.* Based on two studies, the meta-analysis indicated an elevated risk of IHD associated with iso-strain (table 3, supplementary material VIII). The Belstress cohort, which included workers from public and private sectors, found substantially increased risk of IHD among those with high baseline iso-strain. IHD was defined by various clinical endpoints including unstable angina (32). Analyses of multiplicative interaction between demands, control and social support were not provided, but stratified analyses suggested that associations were driven by low social support. These results were only partly consistent with findings in a large national French mortality study, which reported weak associations in both sexes (38).

*Job demands, job control and social support.* The three core components of the JC model were each associated with increasing risk of IHD with risk estimates close to 1.0. The risk increased monotonously with tertile level of job demands in five studies but in no case with a significant trend test (supplementary material IX). Similarly, there were no consistent indications of exposure–response associations for job control or social support (supplementary material X and XI).

## Discussion

In this systematic review addressing causal links between job strain and IHD, we applied predefined and rigorous inclusion criteria regarding study design, exposure and outcome assessment, and statistical analysis. Based on these criteria, we identified 26 high-quality epidemiological studies published in 1994–2023. All were cohort studies except for one case–control study. Job strain was defined and analyzed differently across studies, and across 122 fully adjusted RRE, it was associated with a modest increase in risk of IHD. The association appeared stronger for iso-strain, but this finding was based on only two studies. When examined separately, high psychological demands, low decision latitude, and low social support each showed weak positive associations with IHD risk. Findings based on JEM generally failed to reproduce results derived from self-reported exposures. Furthermore, three studies explicitly testing multiplicative interactions between job demands and control did not find significant interaction effects, questioning the central assumption of the JC model. Very limited data for exposure–response associations were available in studies of job strain, which constitutes a major limitation when evaluating causal inference.

Our findings align broadly with those of earlier systematic reviews (2, 3, 5–10, 13, 49, 50). Eleven previous systematic reviews, including five meta-analyses published since 1999, have similarly reported an association between job strain and IHD, although the magnitude of association has tended to decline as larger, more recent cohort studies have been incorporated (13) This also includes a review restricted to coronary heart disease mortality, which only revealed significant associations with job control (8). Most previous reviews noted limitations related to study design and exposure assessment, and only one concluded that the evidence for a causal relationship was sufficient (50). Findings regarding the separate effects of job demands and job control have been less consistent, and some authors have argued that the relative importance of these dimensions may change over time or differ between occupational groups, particularly between white- and blue-collar workers (49). Despite long-standing calls for intervention studies to establish causality (13), no large-scale workplace trials have yet been completed, reflecting the considerable challenges of conducting such research.

Several methodological factors may have contributed to underestimation of true associations. Limited exposure contrast was common, as few studies targeted highly exposed groups and most employed broad exposure categories that may obscure elevated risks among those with more extreme exposures. Psychosocial exposure scales (eg, on job demands and control) were frequently categorized into tertiles or – less often – into quartiles. As the distribution of these exposures is highly skewed, tertiles may only represent minimal differences in absolute exposure values. Many studies relied on single baseline exposure assessments, neglecting changes or cumulative effects over time. Adjustment for behavioral and medical risk factors – some of which may represent mediating rather than confounding variables – could also have introduced overadjustment bias, thereby attenuating associations. Additionally, healthy worker selection, particularly in samples of older employees, and subjective interpretation of self-report items may have further masked true effects (49).

Conversely, other methodological and conceptual issues could have inflated risk estimates (14). The majority of studies relied on self-reported exposures, which may be influenced by negative affectivity or health-related reporting bias. As Karasek noted already in 1982, individuals with adverse health perceptions may report their work environment more negatively and seek medical attention more frequently, potentially increasing disease detection (51). This interpretation is supported by generally lower risk estimates for cause-specific mortality compared with non-fatal outcomes and by weaker associations in studies using partially independent exposure measures such as JEM. The latter approach is less susceptible to self-report bias but may less accurately pick up some exposures and thus produce bias toward the null. Residual confounding is another important concern, given the modest size of observed associations. Socioeconomic position is strongly linked to both workplace exposures and cardiovascular outcomes (15, 52). Although all included studies adjusted for some measure of socioeconomic status, this may have been insufficient. In one study, an effect of job strain was found when models adjusted for income but not when education was included (39). Few studies considered early-life adversity, social isolation, or other co-exposures at work, leaving the possibility of uncontrolled confounding. Moreover, adjustment for behavioral and medical risk factors typically reduced effect sizes. However, since they were measured concurrently with exposure, it remains unclear whether they act as confounders or mediators. Only three studies examined job strain effects while simultaneously accounting for job demands and control, leaving uncertainty as to which component drives observed associations (36). Selective reporting, particularly in large datasets with multiple comparisons, may also have exaggerated apparent effects (27). Finally, meta-analyses stratified by study size indicated that risk estimates for job strain tended to be weaker in larger studies, which – when considered together with the funnel plot results – may suggest the presence of publication bias leading to inflated meta-analytic risk estimates for smaller studies.

Taken together, numerous sources of bias are likely to have both inflated and attenuated risk estimates, and it is not possible to quantify their net effect size and direction with confidence. The pronounced heterogeneity in findings – across geographical regions, population characteristics, sampling procedures, exposure and outcome definitions, and analytic strategies – remains largely unexplained. This heterogeneity may violate key assumptions for meta-analysis. As such, the pooled estimates and CI should not be interpreted as applying to any real-world population. Instead, they serve as rough indicators of direction and magnitude of effects and must be understood in the context of bias and confounding.

Beyond the methodological considerations, there is indirect but biologically plausible support for causal pathways linking psychosocial work factors to cardiovascular disease. Job strain has been associated with several established cardiovascular risk factors, including hypertension, smoking, obesity, physical inactivity, metabolic syndrome, and dysregulated biomarkers such as blood lipids and heart rate variability (53–56). Acute stress responses may also act as triggers for cardiovascular events, through mechanisms involving sympathetic activation, transient blood pressure elevation, reduced arrhythmic threshold, and pro-inflammatory or procoagulant changes (57). Thus, psychosocial work stressors could contribute to IHD both as chronic determinants and as acute precipitating factors. Nonetheless, empirical demonstration of mediation through these mechanisms remains limited.

We are reluctant to estimate the population attributable fraction of IHD attributable to job strain at the population level because the causal nature of the association remains uncertain and reliable estimates of exposure prevalence are difficult to obtain. Nonetheless, the IPD-Work Consortium has estimated a population attributable fraction of approximately 3–4% (27), yet other researchers have questioned this estimate (58).

The dominance of the demands–control model over recent decades has facilitated comparability across studies but may also have constrained conceptual innovation. Newer approaches that emphasize organizational and structural determinants of work stress (58, 59) offer promise both for research by allowing more objective exposure measures and for practice by identifying modifiable organizational determinants of work-related IHD.

### Concluding remarks

Given the small and inconsistent risk estimates, the potential for multiple biases in both directions, and the absence of robust exposure–response relationships, the current evidence does not substantiate a causal association between job strain and IHD but, simultaneously, it does not exclude the potential for causal associations. Notwithstanding methodological issues that may bias results, upwards and downwards, the findings suggest that any true effect – if present – is likely to be small. Consequently, the current evidence provides only limited guidance for preventive strategies targeting IHD through workplace psychosocial factors.

## Supplementary material

Supplementary material
