# Supplementary material for: The demands–control–support work stress model and risk of ischemic heart disease: causal inference based on observational epidemiology
Source: Scand J Work Environ Health. 2026 Jun 26;52(4):360–70. doi: 10.5271/sjweh.4299 (PMC13344107; doi:10.5271/sjweh.4299)
Supplement: Supplementary material [file SJWEH-52-360-S001.pdf]

The demands–control–support work stress model and risk of ischemic heart disease: causal inference based on observational epidemiology[1]

by Jens Peter Bonde, MD, DMsc,[2] Stinna Skaaby, MD, PhD, Esben M Flachs, PhD, Maureen Dollard, PhD, Katherine Keyes, PhD, Annika Rosengren, MD, PhD, Ingrid S Mehlum, MD, PhD, Sigurd Mikkelsen, MD, DMsc

1. SUPPLEMENTAL MATERIAL
2. Correspondence to: Jens Peter Bonde, Department of Occupational and Environmental Medicine, Bispebjerg and Frederiksberg University Hospital, Copenhagen, Denmark. [E-mail: Jens.peter.ellekilde.bonde@regionh.dk]

I PubMed and Embase Literature Search. 2
II Flow diagram on article selection 3
III Characteristics of included studies 4
IV Reasons for exclusion of full text read papers 20
V Studies by type of exposure 28
VI Covariates 29
VII Funnel plot job strain and ischemic heart disease 30
VIII Forest plot of high isostrain and ischemic heart disease 31
IX Forest plot of high job demands and ischemic heart disease 32
X Forest plot of low job control and ischemic heart disease 33
XI Forest plot of low workplace social support and ischemic heart disease. 34
XII List of abbreviations in tables and forest plots 35

I PubMed and Embase Literature Search.

PubMed 15.11.2024		
Search	Query	Results
#1<br>Design	cohort studies[MeSH] OR prospective studies[MeSH] OR longitudinal studies[MeSH] OR cross over studies[MeSH] OR controlled clinical trial[Publication Type] OR cohort[Tiab] OR prospective[Tiab] OR longitudinal[Tiab] OR cross sectional studies[MeSH] OR case-control studies[MeSH] OR cross-sectional[Tiab] OR cross sectional[Tiab] OR case-control[Tiab] OR case control[Tiab] OR case-referent[Tiab] OR case referent[Tiab] OR surveys and questionnaires/epidemiology[MeSH] OR survey[Tiab] OR case-crossover[Tiab] OR case crossover[Tiab] OR case-only[Tiab] OR intervention[Tiab] OR job exposure matrix[tiab] OR job-exposure matrix[tiab] or JEM[tiab] Filters: English, Humans, Adult: 19+ years	2,969,381
#2<br>Exposure,<br>MeSH	occupational stress [Mesh] OR social capital[MeSH] OR job security[Mesh] OR job satisfaction[Mesh] OR occupational violence[Mesh] OR occupational bullying[Mesh] NOT editorial[publication type] NOT letter[publication type] NOT review[publication type] NOT systematic review[publication type] Filters: English, Humans, Adult: 19+ years	19,671
#3<br>Exposure,<br>free text	psychosocial stress*[tiab] OR psychosocial factor*[tiab] OR psychosocial risk*[tiab] OR stress at work[tiab] OR work stress*[tiab] OR occupational stress*[tiab] OR work environment[tiab] OR occupational strain[tiab] OR job characteristic*[tiab] OR job stress*[tiab] OR job strain[tiab] OR job demand*[tiab] OR job control[tiab] OR decision latitude[tiab] OR decision authority[tiab] OR skill discretion[tiab] OR social support[tiab] OR isostrain[tiab] OR workload[tiab] OR time pressure[tiab] OR overtime[tiab] OR hour spent working[tiab] OR emotional demand*[tiab] OR care giver*[tiab] OR social capital[tiab] OR effort-reward imbalance[tiab] OR overcommitment[tiab] OR job insecurity[tiab] OR job security[Tiab] OR downsizing[tiab] OR working hour*[tiab] OR excessive work[tiab] OR sedentary work[tiab] OR violence[tiab] OR bullying[tiab] OR harassment[tiab] OR psychosocial resource*[tiab] OR justice[tiab] OR injustice[tiab] NOT editorial[publication type] NOT letter[publication type] NOT review[publication type] NOT systematic review[publication type] Filters: English, Humans, Adult: 19+ years	99,814
#4<br>Outcome<br>MeSH	Myocardial ischemia[Mesh] OR Stroke[Mesh] NOT editorial[publication type] NOT letter[publication type] NOT review[publication type] NOT systematic review[publication type] Filters: English, Humans, Adult: 19+ years	265,693
#5<br>Outcome,<br>Free text	heart disease*[ti] OR cardiovascular disease*[tiab] OR cardiovascular event*[ti] OR cardiovascular system[ti] OR cardiovascular outcome*[ti] OR cardiovascular mortality[ti] OR all-cause mortality[ti] OR coronary heart disease*[ti] OR myocardial infarction[tiab] OR coronary event*[ti] OR coronary health[ti] OR stroke[ti] OR cerebrovascular disease*[ti] NOT editorial[publication type] NOT letter[publication type] NOT review[publication type] NOT systematic review[publication type] Filters: English, Humans, Adult: 19+ years	246,260
#6	#2 OR #3 (exposure)	111,183
#7	#4 OR #5 (outcome)	372,122
#8	#6 AND #7 (exposure and outcome)	3893
#9	#8 AND #1 (exposure and outcome and study design)	2744

**Embase 16.11.2024**		
Search	Query	Results
#1<br>Exposure	('psychosocial stress' or 'psychosocial factor' or 'psychosocial risk' or 'stress at work' or 'work stress' or 'occupational stress' or 'work environment' or 'occupational strain' or 'job characteristic' or 'job stress' or 'job strain' or 'job demand' or 'job control' or 'decision latitude' or 'decision authority' or 'skill discretion' or 'social support' or 'isostrain' or 'workload' or 'time pressure' or 'overtime' or 'hour spent working' or 'emotional demand' or 'care giver' or 'social capital' or 'effort-reward imbalance' or 'overcommitment' or 'job insecurity' or 'job security' or 'downsizing' or 'working hour' or 'excessive work' or 'sedentary work' or 'violence' or 'bullying' or 'harassment' or 'psychosocial resource' or 'justice' or 'injustice').ti,kw	110,819
#2<br>Outcome	('ischemic hear disease' or 'stroke' or 'heart disease' or 'cardiovascular disease' or 'cardiovascular event' or 'cardiovascular system' or 'cardiovascular outcome' or 'cardiovascular mortality' or 'all-cause mortality' or 'coronary heart disease' or 'myocardial infarction' or 'coronary event' or 'coronary health' or 'cerebrovascular disease').me,ti,ab.	1,987,486
#3<br>Exposure and<br>outcome	#1 AND #2	2876

#4	limit 3 to (human and english language)	2552
#5	limit 4 to "remove medline records"	**675**

II Flow diagram on article selection

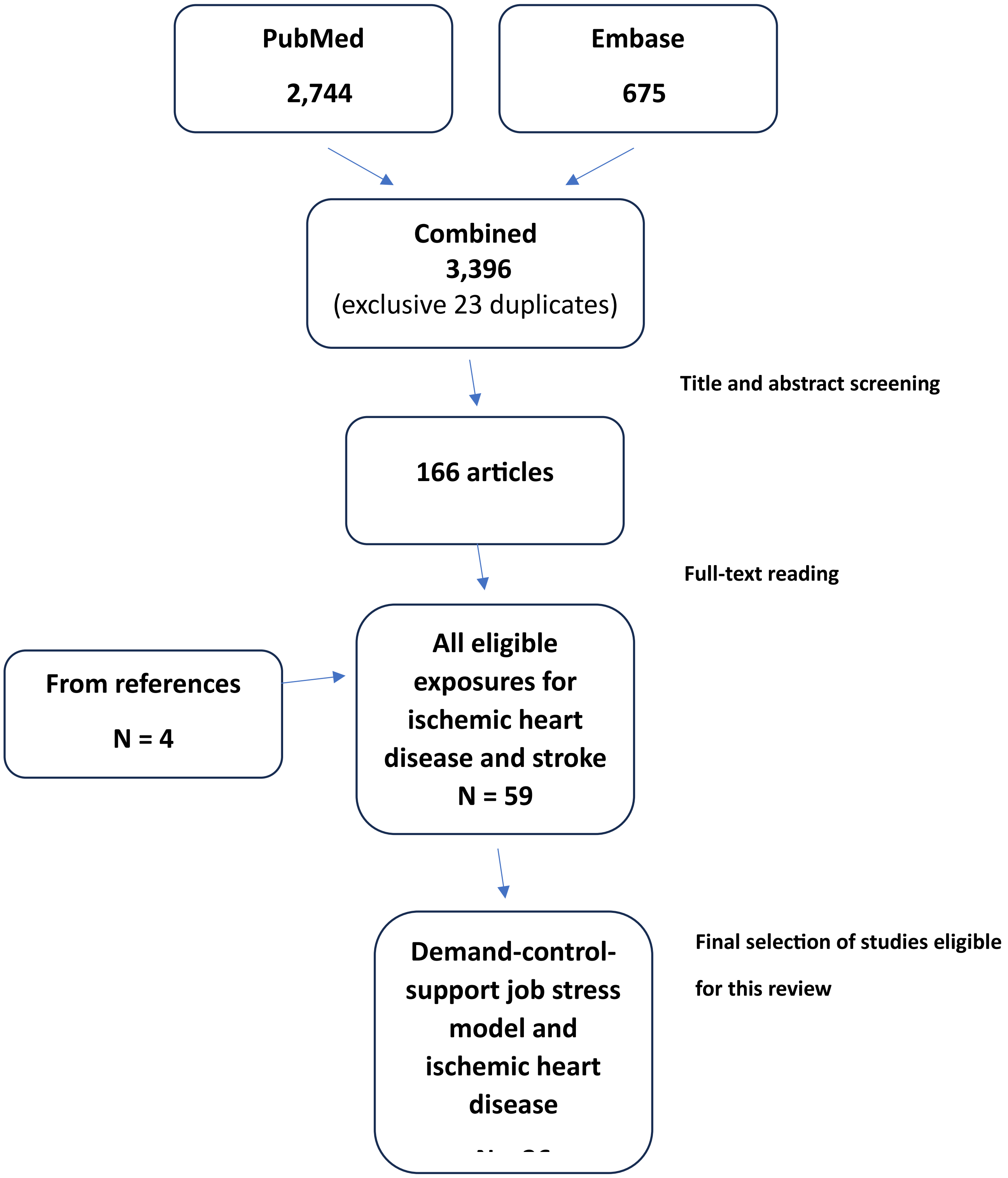

III Characteristics of included studies

FIRST AUTHOR year (study label)	COUNTRY	POPULATION	N	WO-MEN %	FOLLOW-UP Period	FOLLOW-UP Duration	OUTCOME Type Ascertainment	N cases	EXPOSURE Ascertainment Contrast	STRATA	MINIMALLY ADJUSTED RR	MINIMALLY ADJUSTED 95% CI	FULLY ADJUSTED RR	FULLY ADJUSTED 95% CI
**FOLLOW-UP STUDIES ISCHEMIC HEART DISEASE**														
Alterman 1994 (Western Electric Study)	USA	Male middle-aged employees at the Hawthorne Works, Chicago	1,683	0	1957-1983	25.0	**IHD mortality** Death registry	283	JEM survey **Job demands** 10% increase in job-demand item sum scores	All	0.79	0.48-1.28	0.76	0.55-1.05
									**Decision latitude:** 20% increase in job control item sum scores	All	0.76	0.59-0.97	0.85	0.70-1.03
									**Job strain: highest strain vs lowest strain, tertile split**	All	1.48	0.98-2.24	1.03	0.75-1.41
Steenland 1997 (NHANES)	USA	Male employees aged 25-74	3,575	0	1971-1987	NR	**IHD** ICD-9 410-414 Hospital discharge diagnoses and death certificates	519	JEM, survey based multi adjusted job scores. Job titles from 1970 US census					
									**Job demands:** quartiles of item sum scores, higher vs lowest	**White collar** 2nd quartile			0.69	0.45-1.06
										3rd quartile			1.09	0.73-1.64
										4th quartile			0.93	0.61-1.44
										**Blue collar** 2nd quartile			0.83	0.62-1.12
										3rdquartile			0.83	0.58-1.18
										4th quartile			0.40	0.64-1.03
									**Job control:** quartiles of item sum scores, higher vs lowest	**White collar** 2nd quartile			0.71	0.40-1.28

III Characteristics of included studies

FIRST AUTHOR year (study label)	COUNTRY	POPULATION	N	WO-MEN %	FOLLOW-UP Period	FOLLOW-UP Dura-tion	OUTCOME Type Ascertainment	N cases	EXPOSURE Ascertainment Contrast	STRATA	MINIMALLY ADJUSTED RR	MINIMALLY ADJUSTED 95% CI	FULLY ADJUSTED RR	FULLY ADJUSTED 95% CI
										3rd quartile			0.97	0.57-1.65
										4thquartile			0.74	0.43-1.26
										**Blue collar** 2nd quartile			0.87	0.66-1.16
										3rd quartile			0.67	0.48-0.93
										4th quartile			0.69	0.46-1.02
									**Job strain,** quadrant, median split, strain vs relaxed	White collar			1.05	0.63-1.77
										Blue collar			1.14	0.80-1.63
Lee 2002 (Nurses' Health Study)	USA	Registered nurses in a health study	35,038	100	1992-1996	4.0	**MI and fatal IHD** Review of medical records and death certificates after reported CVD events	146	Baseline selfAQ **Job demands** Tertiles of item sum scores	Lowest tertile	1	reference	1	reference
										Intermediate tertile	1.35	0.93-1.97	1.32	0.90-1.93
										Highest tertile	0.85	0.55-1.32	0.8	0.52-1.24
									**Decision latitude** Tertiles of item sum scores	Highest tertile	1	reference	1	reference
										Intermediate tertile	0.85	0.57-1.28	0.81	0.54-1.22
										Lowest tertile	1.06	0.72-1.58	0.97	0.65-1.45
									**Job strain** Quadrant, median split, strain vs relaxed	All	0.80	0.48-1.34	0.71	0.42-1.19

III Characteristics of included studies

FIRST AUTHOR year (study label)	COUNTRY	POPULATION	N	WO-MEN %	FOLLOW-UP		OUTCOME Type Ascertainment	N cases	EXPOSURE Ascertainment Contrast	STRATA	MINIMALLY ADJUSTED		FULLY ADJUSTED	
					Period	Dura-tion					RR	95% CI	RR	95% CI
									**Social support** Median split, low vs high	All	1.28	0.90-1.83	1.15	0.80-1.64
Virtanen 2002	Finland	Finish census files including men 25-65 in 1980 with the same occupation 1975 and 1980	Approxi-mately 386.000	0	1981-94	14.0	**MI mortality** National death registry	8,378	JEM expert **Job control** Low vs medium and high (no details)	Men	nr	-	1.11	1.04-1.19
									**Work load** Medium vs low (no details)	Men	nr	-	1.05	0.97-1.13
									**Work load** High vs low	Men	nr	-	1.13	0.96-1.33
Kuper 2003 (Whitehall II)	UK	Civil servants in offices in 20 departments in London	10,308	33	1985-2000	11.0	**MI and fatal IHD** Medical record review of reported events	296	Baseline SelfAQ **Job demands** Tertiles of item sum scores	**Men** Lowest tertile	1	reference	1	reference
										Intermediate tertile	1.49	1.06-2.10	1.33	0.93-1.90
										Highest tertile	1.22	0.84-1.78	1.17	0.79-1.73
										**Women** Lowest tertile	1	reference	1	reference
										Intermediate tertile	1.30	0.73-2.30	1.31	0.71-2.40
										Highest tertile	1.37	0.67-2.80	1.85	0.89-3.85
									**Decision latitude** Tertiles of item sum scores	**Men** Highest tertile	1	reference	1	reference
										Intermediate tertile	1.32	0.99-1.76	1.32	0.97-1.79

III Characteristics of included studies

FIRST AUTHOR year (study label)	COUNTRY	POPULATION	N	WO-MEN %	FOLLOW-UP		OUTCOME Type Ascertainment	N cases	EXPOSURE Ascertainment Contrast	STRATA	MINIMALLY ADJUSTED		FULLY ADJUSTED	
					Period	Dura-tion					RR	95% CI	RR	95% CI
										Lowest tertile	1.14	0.82-1.59	1.01	0.70-1.45
										**Women**<br>Highest tertile	1	reference	1	reference
										Intermediate tertile	0.68	0.30-1.55	0.70	0.30-1.64
										Lowest Q3	1.06	0.53-2.08	0.92	0.45-0.89
									**Job strain**<br>Quadrant, median split, strain vs relaxed	All	1.42	0.99-2.05	1.16	0.78-1.71
Eaker 2004 (Framingham Offspring Study)	USA	Offspring (and spouses) from a population-based study	3,039	44	1984-99	10.0	**IHD and fatal IHD**<br>Scheduled clinical examinations during follow up, strict criteria. Death certificates	149	Baseline SelfAQ<br>**Job strain**<br>Quadrant, median split, strain vs relaxed (low strain)	Men	NR		1.18	0.69-2.00
										Women	NR		0.61	0.21-1.75
									**Job demands**<br>Risk by one SD increase in item sum score	Men	NR		1	0.97-1.04
										Women	NR		0.99	0.93-1.06
									**Decision latitude**<br>Risk by one SD increase in item sum score	Men	NR		1	0.95-1.06
										Women	NR		1.85	1.21-2.85
De Bacquer 2005 (BELSTRESS)	Belgium	Employees in 25 large companies and public administration s across Belgium	14,337	0	1994-1999	3.2	**IHD**<br>Medically ascertained acute myocardial infarction, unstable angina, coronary artery bypass or coronary	87	Baseline SelfAQ<br>**Job demands,**<br>Tertiles of item sum scores	Lowest tertile	1	reference	1	reference
										Intermediate tertile	1.14	0.68-1.90	1.26	0.73-2.14

III Characteristics of included studies

FIRST AUTHOR year (study label)	COUNTRY	POPULATION	N	WO-MEN %	FOLLOW-UP Period	FOLLOW-UP Duration	OUTCOME Type Ascertainment	N cases	EXPOSURE Ascertainment Contrast	STRATA	MINIMALLY ADJUSTED RR	MINIMALLY ADJUSTED 95% CI	FULLY ADJUSTED RR	FULLY ADJUSTED 95% CI
							angioplasty at baseline clinical examination			Highest tertile	1.31	0.77-2.24	1.43	0.80-2.57
									**Decision latitude** Tertiles of item sum scores	Highest tertile	1	reference	1	reference
										Intermediate tertile	0.91	0.55-1.52	0.73	0.42-1.26
										Lowest tertile	1	0.60-1.65	0.83	0.48-1.43
									**Social support,** two subscales, co-worker and supervisor support Tertiles of item sum scores	Highest tertiles	1	reference	1	reference
										Intermediate tertiles	1.36	0.79-2.32	1.58	0.91-2.74
										Lowest tertiles	2.11	1.27-3.52	2.36	1.38-4.01
									**Job strain**, quadrant, median split, strain vs relaxed	All	1.35	0.73-2.49	1.26	0.66-2.41
									**Isostrain**, quadrant, median split, high iso vs low	All	1.91	1.07-3.41	1.92	1.05-3.53
Kuper 2006	Sweden	Women in the Uppsala Health care region enrolled into a cohort study	48,066	100	1991-2002	11.0	**MI and fatal IHD** Registry based medical ascertainment	210	Baseline SelfAQ **Job demands** Tertiles of item sum scores	Lowest tertile	1	reference	1	Reference
										Intermediate tertile	0.9	0.5-1.6	0.8	0.4-1.5
										Highest tertile	1.4	0.9-2.3	1.4	0.8-2.3

III Characteristics of included studies

FIRST AUTHOR year (study label)	COUNTRY	POPULATION	N	WO-MEN %	FOLLOW-UP		OUTCOME Type Ascertainment	N cases	EXPOSURE Ascertainment Contrast	STRATA	MINIMALLY ADJUSTED		FULLY ADJUSTED	
					Period	Dura-tion					RR	95% CI	RR	95% CI
									**Decision latitude,** Tertiles of item sum scores	Highest tertile	1	reference	1	reference
										Intermediate tertile	0.8	0.5-1.4	0.8	0.5-1.4
										Lowest tertile	1	0.6-1.6	0.7	0.4-1.2
									**Social support,** two subscales, co-worker and supervisor support. Tertiles of item sum scores	Highest tertile	1	reference	1	reference
										Intermediate tertile	0.8	0.5-1.4	1	0.5-1.7
										Lowest tertile	1.3	0.8-2.0	1.2	0.7-1.2
									**Job strain,** quadrant, median split, strain vs relaxed	All	1.4	0.7-2.7	1	0.5-1.9
Bonde 2009	Denmark	A cohort of public service employees at Aarhus County and Aarhus municipality identified from electronic company records	18,258	79	2002-2007	5.0	**IHD** National patient registry, ischemic heart disease ICD-8: 410-414; ICD-10: I21, I22 and I24	101	Workunit, baseline SelfAQ **Job demands** Each employee assigned mean scores of the work unit (1106 work units). Quartiles of work unit average sum score	Lowest Q4	NR		1	Reference
										Q2 and Q3			1.2	0.7-1.9
										Highest Q1			1.3	0.8-2.3
									**Job control** Quartiles of work unit average sum score	Lowest Q4	NR		2	1.1-3.6
										Q2 and Q3			1.4	0.8-2.4

III Characteristics of included studies

FIRST AUTHOR year (study label)	COUNTRY	POPULATION	N	WO-MEN %	FOLLOW-UP		OUTCOME Type Ascertainment	N cases	EXPOSURE Ascertainment Contrast	STRATA	MINIMALLY ADJUSTED		FULLY ADJUSTED	
					Period	Dura-tion					RR	95% CI	RR	95% CI
										Highest Q1			1	Reference
									**Social support at work** Quartiles of work unit average sum score	Lowest Q4	NR		1.1	0.6-2.0
										Q2 and Q4_3			1	0.6-1.7
										Highest Q1			1	reference
									**Job strain** Quadrant, median, strain vs all others	All	NR		1.3	0.9-2.1
Netterstrøm 2010 (Copenhagen City Heart Study)	Denmark	Employed citizens aged 30+ in Copenhagen City	1,141	52	1992-2007	13-15	**IHD or fatal IHD** Patient and death registry, ICD-10 I20-25 or equivalent in ICD-8	IHD 104 MI 49	Baseline SelfAQ 27 items, 3 response categories (often/ sometimes/rarely) **Job demands** High vs low, split unknown	Men	1.1	0.6-1.8	1.5	0.8-2.6
										Women	0.9	0.4-1.8	1.1	0.5-1.3
									**Control** High vs low, split unknown	Men	0.7	0.4-1.1	0.9	0.5-1.3
										Women	0.6	0.3-1.4	1.2	0.5-3.1
									**Social support** High vs low, split unknown	Men	0.8	0.5-1.4	0.8	0.5-1.4
										Women	0.6	0.3-1.4	0.9	0.4-1.4
									**Job strain** Quadrant, split unknown, yes/no	Men	1.3	0.8-2.1	1.6	0.7-3.7
										Women	1.2	0.6-2.3	1.1	0.3-4.2

III Characteristics of included studies

FIRST AUTHOR year (study label)	COUNTRY	POPULATION	N	WO-MEN %	FOLLOW-UP Period	FOLLOW-UP Duration	OUTCOME Type Ascertainment	N cases	EXPOSURE Ascertainment Contrast	STRATA	MINIMALLY ADJUSTED RR	MINIMALLY ADJUSTED 95% CI	FULLY ADJUSTED RR	FULLY ADJUSTED 95% CI
Kivimäki 2012 (IPD Consortium)	7 European countries	Employees in one of 13 cohorts of the IPD consortium	197,473	49	1985-2006	8.0	**MI and fatal MI** Hospital records for admission for non-fatal MI (ICD-10: I21-I22; ICD – 8: 410) or death registries for fatal CHD	2,358	Baseline SelfAQ **Job strain** Quadrant, study specific median, strain vs all other	All	1.23	1.10-1.37	1.17	1.05 -1.31
Slopen 2012 (Women's Health Study)	USA	Women in health-related occupations participating in a study addressing effects of Aspirin	22,086	100	1998-2007	10.0	**MI or IHD** Self-reported cases of non-fatal myocardial infarction, coronary revascularization or ischemic stroke verified by review of medical records. Fatal CVD, death registry	170	Baseline SelfAQ **Job strain** High demands and low control, quadrant method, median split, high strain vs low strain	MI	1.88	1.18-3.01	1.67	1.04-2.70
								440		Coronary revasc.	1.59	1.19-2.13	1.41	1.05-1.90
Torén 2014	Sweden	Population based cohort in Gothenburg born 1915-1925	6,070	0	1975-2000	16.0	**MI** Hospital discharge and death registry-es. Non-fatal MI ICD-10 I21 Fatal CHD ICD-10 I20-I25	1,052	JEM survey-based **Job demands** Median split of item sum scores. High vs low	MI White collar	1.00	0.80-1.26	NR	NR
										MI Blue collar	1.15	0.93-1.41	NR	NR

III Characteristics of included studies

FIRST AUTHOR year (study label)	COUNTRY	POPULATION	N	WO-MEN %	FOLLOW-UP Period	FOLLOW-UP Dura-tion	OUTCOME Type Ascertainment	N cases	EXPOSURE Ascertainment Contrast	STRATA	MINIMALLY ADJUSTED RR	MINIMALLY ADJUSTED 95% CI	FULLY ADJUSTED RR	FULLY ADJUSTED 95% CI
									**Job control**<br>Median split of item sum scores. Low vs high	MI<br>White collar	1.06	0.84-1.33	NR	NR
										MI<br>Blue collar	1.23	0.98-1.56	NR	NR
									**Job strain**<br>Quadrant method, median split, high strain vs relaxed (no strain)	MI<br>White collar	1.15	0.69-1.92	NR	NR
										MI<br>Blue collar	1.36	1.01-1.84	NR	NR
Schiöler 2015	Sweden	A cohort of male construction workers established 1989-93	75,236	0	1989-2004	12.6	**Fatal and non-fatal IHD.**<br>Hospitalization for MI (ICD-9 410 or ICD-10 I21 or CHD death (ICD-10 I20-I25)	CHD 1,884<br>Stroke 739	Baseline SelfAQ<br>Job content questionnaire using ad hoc questions examined by factor analysis.<br>**Job demands**<br>4 item scale with 5 response levels. Item sum scores divided into quintiles; highest vs lowest	MI and IHD death	1.15	0.99-1.33	1.18	1.02-1.37
									**Job control**<br>3 items, 5 response levels. Item sum scores divided into quintiles, lowest vs highest	MI and IHD death	1.12	0.97-129	1.13	0.98-1.31

III Characteristics of included studies

FIRST AUTHOR year (study label)	COUNTRY	POPULATION	N	WO-MEN %	FOLLOW-UP Period	Dura-tion	OUTCOME Type Ascertainment	N cases	EXPOSURE Ascertainment Contrast	STRATA	MINIMALLY ADJUSTED RR	95% CI	FULLY ADJUSTED RR	95% CI
									**Social support** 2 items, 5 response scale. Item sum scores divided into quintiles, lowest vs highest	MI and IHD death	0.98	0.86-1.11	1.01	0.89-1.15
									**Jobstrain**: quintiles, quintile split, highest vs lowest	MI and IHD death	1.10	0.89-1.35	1.07	0.87-1.33
Ferrario, 2017	Italy	Employees in 3 population-based and one factory-based cohort north of Milan	3,284	0	1989-2008	14.6	**Fatal and non-fatal IHD** Medically verified fatal and non-fatal acute coronary events (AMI, coronary syndrome, revascularization)	172	SelfAQ using modified JCQ. **Jobstrain** Quadrant, median split, strain vs all others (no strain)	Non-manual workers	NR		1.70	0.94-3.08
										Manual workers	NR		1.94	1.13-3.32
Power, 2019	Canada	Population-based cohort of employed residents 40-60 years old in Quebec recruited through a public health insurance database	8,073	50	2009	6.6	**IHD** Diagnoses from insurance billing system: ICD-9: 410-414 or ICD-10: I21 and I23	557	Baseline SelfAQ **Job strain** JCQ 10-item version. Job strain defined by the ratio of demands and inversed job control item sum scores. Ratio >= 1.0 vs < 1.0	Men	1.22	0.81-1.84	0.96	0.62-1.49
										Women	1.85	1.19-2.90	1.63	1.02-2.60

III Characteristics of included studies

FIRST AUTHOR year (study label)	COUNTRY	POPULATION	N	WO-MEN %	FOLLOW-UP Period	FOLLOW-UP Dura-tion	OUTCOME Type Ascertainment	N cases	EXPOSURE Ascertainment Contrast	STRATA	MINIMALLY ADJUSTED RR	MINIMALLY ADJUSTED 95% CI	FULLY ADJUSTED RR	FULLY ADJUSTED 95% CI
Niedhammer 2020	France	National representative cohort of employees	1,496,332	47	1976-2002	17.0	**IHD mortality on the job** French national death registry: Deaths ischemic heart disease ICD-10 I20-I25 and equivalent for ICD-8 and 9.	IHD 1551 Stroke 689	JEM survey Sex, age, job title, industry and company size specific JEM based upon occupational JCQ surveys. **Job demands.** Recency weighted cumulative exposure. High psychological demands vs low demands (median split)	IHD mortality on the job Men	NR		1.06	0.92-1.23
										Women			0.92	0.56-1.51
									**Decision latitude**. Recency weighted cumulative exposure low control vs high control (median split)	IHD mortality on the job Men	NR		1.14	0.98-1.32
										Women			1.26	0.67-2.39
									**Social support.** Recency weighted cumulative exposure low support vs high support (median split)	IHD mortality on the job Men	NR		1.16	1.00-1.34
										Women			1.58	0.92-2.72
									**Job strain.** Recency weighted cumulative exposure to low control and high demands vs high control and low demand (median split)	IHD mortality on the job Men	NR		1.16	0.97-1.38
										Women			1.15	0.70-1.89

III Characteristics of included studies

FIRST AUTHOR year (study label)	COUNTRY	POPULATION	N	WO-MEN %	FOLLOW-UP Period	FOLLOW-UP Dura-tion	OUTCOME Type Ascertainment	N cases	EXPOSURE Ascertainment Contrast	STRATA	MINIMALLY ADJUSTED RR	MINIMALLY ADJUSTED 95% CI	FULLY ADJUSTED RR	FULLY ADJUSTED 95% CI
									**Job isotrain**. Recency weighted cumulative exposure to high demands, low control and low support vs low demands, high control and high support	IHD mortality on the job Men	NR--		1.14	0.95-1.37
										Women			1.13	0.68-1.87
Rugulies 2020	Denmark	Employed residents in Denmark 2000	1,660,150	49	2001-2010	10.0	**Fatal and non-fatal MI** National patient registry, myocardial infarction ICD-10 I21 and I22 and CHD mortality (ICD-10 I20-I25)	24,159	JEM survey based. Sex-, age- and calendar year specific JEM assigning work environment survey sum scores for questions on job demands (3 items) and job control (5 items). **Job strain** quadrant, median splits of item sum scores, strain vs relaxed	All	1.17	1.14-1.20	1.00	0.98-1.03
Smith 2021	Canada	Employees participating in the nationwide Canadian Community Health Surveys 2000-2003	13,291	48	2001-2017	~15	**MI and congestive heart failure** diagnoses retrieved from two medical databases	641 (calc. from inciden-ce rates)	Baseline interview convenience questions. **Job demands**, 2 items, quartiles of item sum scores, highest vs lowest	Men	1.05	0.63-1.78	1.10	0.67-1.83
										Women	0.62	0.30-1.28	0.60	0.29-1.24
									**Job control,** 5 items, quartiles of item sum scores, lowest vs highest	Men	0.73	0.42-1.27	0.72	0.42-1.24
										Women	0.85	0.46-1.56	0.78	0.42-1.43

III Characteristics of included studies

FIRST AUTHOR year (study label)	COUNTRY	POPULATION	N	WO-MEN %	FOLLOW-UP		OUTCOME Type Ascertainment	N cases	EXPOSURE Ascertainment Contrast	STRATA	MINIMALLY ADJUSTED		FULLY ADJUSTED	
					Period	Dura-tion					RR	95% CI	RR	95% CI
									**Job strain** quadrant, median split, strain vs no strain (relaxed)	Men	0.83	0.50-1.37	0.82	0.50-1.36
										Women	0.89	0.46-1.73	0.86	0.45-1.65
Wang 2021	USA	Postmenopausal women recruited from 40 clinical centers across USA	80825	100	1993-2013	15.0	**MI and IHD mortality.** Medical review of annually self-reported CHD event for diagnosis of MI and IHD mortality analyzed as a pooled outcome	3,841	JEM based upon independent review of job titles with five items for job demands (different from JCQ) and 8 for job control. **Demand,** median item sum scores, high vs low	Current workers to retirement	0.90	0.81-0.99	0.96	0.91-1.02
									**Control,** median item sum scores, high vs low	Current workers to retirement	0.81	0.74-0.89	0.97	0.92-1.03
									**Jobstrain,** quadrant, median splits strain vs relaxed	Current workers to retirement	1.12	0.95-1.32	1.01	0.91-1.10
Lavigne-Robichaud 2023	Canada	White-collar employees in 19 public and semi-public enterprises in the region of Quebec	6,465	52	2000-2018	17.0	**IHD.** Medico-administrative database diagnoses ICD-9: 410-414; ICD-10: I20-I25 (specificity 97.5%)	836	Baseline SelfAQ French version of job content questionnaire (18 items, 4-point Likert scale for demands and control). **Job strain,** quadrant, medium split, strain vs relaxed. Medians based upon general Quebec working population study.	Men<br>Women	1.45<br>1.08	1.01-2.10<br>0.65-1.78	1.47<br>1.02	1.09 -1.99<br>0.62-1.67

III Characteristics of included studies

FIRST AUTHOR year (study label)	COUNTRY	POPULATION	N	WO-MEN %	FOLLOW-UP Period	Dura-tion	OUTCOME Type Ascertainment	N cases	EXPOSURE Ascertainment Contrast	STRATA	MINIMALLY ADJUSTED RR	95% CI	FULLY ADJUSTED RR	95% CI
**Case-control studies**														
Hammar 1998	Sweden	Residents in Stockholm County and 4 rural Swedish counties	Cases	10,008	1976-84	11.8	**AMI** Cases of first myocardial infarction through hospital and death registries. Two random controls matched by sex, age and year of coronary event.		JEM based on national occupational interview surveys 1977-1979 linked with census data on job titles.					
			Controls	28,448		12.4			**Job demands** 2 items (hectic and psychologically demanding), median of item sum scores, high vs low	Men	0.94	0.89-0.99	nr	nr
										Women	0.95	0.82-1.10	nr	nr
									**Decision latitude** 11 items, median of item sum scores, low vs high	Men	1.19	1.13-1.25	nr	nr
										Women	1.44	1.25-1.65	nr	nr
									**Job strain** Quadrant, median split, high vs low	Men	1.21	1.08-1.35	1.12	0.99-1.27
										Women	1.23	1.01-1.51	1.09	0.82-1.44

ALPHABETIC LIST WITH FULL REFERENCES OF INCLUDED STUDIES

Alterman, T., R. B. Shekelle, S. W. Vernon and K. D. Burau (1994). "Decision latitude, psychologic demand, job strain, and coronary heart disease in the Western Electric Study." Am J Epidemiol **139**(6): 620-627.

Bonde, J. P., T. Munch-Hansen, E. Agerbo, P. Suadicani, J. Wieclaw and N. Westergaard-Nielsen (2009). "Job strain and ischemic heart disease: a prospective study using a new approach for exposure assessment." J Occup Environ Med **51**(6): 732-738.

De Bacquer, D., E. Pelfrene, E. Clays, R. Mak, M. Moreau, P. de Smet, M. Kornitzer and G. De Backer (2005). "Perceived job stress and incidence of coronary events: 3-year follow-up of the Belgian Job Stress Project cohort." Am J Epidemiol **161**(5): 434-441.

Eaker, E. D., L. M. Sullivan, M. Kelly-Hayes, R. B. D'Agostino, Sr. and E. J. Benjamin (2004). "Does job strain increase the risk for coronary heart disease or death in men and women? The Framingham Offspring Study." Am J Epidemiol **159**(10): 950-958.

Ferrario, M. M., G. Veronesi, M. Roncaioli, A. Holtermann, N. Krause, E. Clays, R. Borchini, G. Grassi and G. Cesana (2019). "Exploring the interplay between job strain and different domains of physical activity on the incidence of coronary heart disease in adult men." Eur J Prev Cardiol **26**(17): 1877-1885.

Hammar, N., L. Alfredsson and J. V. Johnson (1998). "Job strain, social support at work, and incidence of myocardial infarction." Occup Environ Med **55**(8): 548-553.

Hemmingsson, T. and I. Lundberg (2006). "Is the association between low job control and coronary heart disease confounded by risk factors measured in childhood and adolescence among Swedish males 40-53 years of age?" Int J Epidemiol **35**(3): 616-622.

Kivimäki, M., S. T. Nyberg, G. D. Batty, E. I. Fransson, K. Heikkilä, L. Alfredsson, J. B. Bjorner, M. Borritz, H. Burr, A. Casini, E. Clays, D. De Bacquer, N. Dragano, J. E. Ferrie, G. A. Geuskens, M. Goldberg, M. Hamer, W. E. Hooftman, I. L. Houtman, M. Joensuu, M. Jokela, F. Kittel, A. Knutsson, M. Koskenvuo, A. Koskinen, A. Kouvonen, M. Kumari, I. E. Madsen, M. G. Marmot, M. L. Nielsen, M. Nordin, T. Oksanen, J. Pentti, R. Rugulies, P. Salo, J. Siegrist, A. Singh-Manoux, S. B. Suominen, A. Väänänen, J. Vahtera, M. Virtanen, P. J. Westerholm, H. Westerlund, M. Zins, A. Steptoe and T. Theorell (2012). "Job strain as a risk factor for coronary heart disease: a collaborative meta-analysis of individual participant data." Lancet **380**(9852): 1491-1497.

Kuper, H., H. O. Adami, T. Theorell and E. Weiderpass (2006). "Psychosocial determinants of coronary heart disease in middle-aged women: a prospective study in Sweden." Am J Epidemiol **164**(4): 349-357.

Kuper, H. and M. Marmot (2003). "Job strain, job demands, decision latitude, and risk of coronary heart disease within the Whitehall II study." J Epidemiol Community Health **57**(2): 147-153.

Lavigne-Robichaud, M., X. Trudel, D. Talbot, A. Milot, M. Gilbert-Ouimet, M. Vézina, D. Laurin, C. E. Dionne, N. Pearce, G. R. Dagenais and C. Brisson (2023). "Psychosocial Stressors at Work and Coronary Heart Disease Risk in Men and Women: 18-Year Prospective Cohort Study of Combined Exposures." Circ Cardiovasc Qual Outcomes **16**(10): e009700.

Lee, S., G. Colditz, L. Berkman and I. Kawachi (2002). "A prospective study of job strain and coronary heart disease in US women." Int J Epidemiol **31**(6): 1147-1153; discussion 1154.

Netterstrøm, B., T. S. Kristensen, G. Jensen and P. Schnor (2010). "Is the demand-control model still a usefull tool to assess work-related psychosocial risk for ischemic heart disease? Results from 14 year follow up in the Copenhagen City Heart study." Int J Occup Med Environ Health **23**(3): 217-224.

Niedhammer, I., A. Milner, B. Geoffroy-Perez, T. Coutrot, A. D. LaMontagne and J. F. Chastang (2020). "Psychosocial work exposures of the job strain model and cardiovascular mortality in France: results from the STRESSJEM prospective study." Scand J Work Environ Health **46**(5): 542-551.

Power, N., S. S. Deschênes, F. Ferri and N. Schmitz (2020). "Job strain and the incidence of heart diseases: A prospective community study in Quebec, Canada." J Psychosom Res **139**: 110268.

Rugulies, R., E. Framke, J. K. Sørensen, A. C. Svane-Petersen, K. Alexanderson, J. P. Bonde, K. Farrants, E. M. Flachs, L. L. Magnusson Hanson, S. T. Nyberg, M. Kivimäki and I. E. Madsen (2020). "Persistent and changing job strain and risk of coronary heart disease. A population-based cohort study of 1.6 million employees in Denmark." Scand J Work Environ Health **46**(5): 498-507.

Schiöler, L., M. Söderberg, A. Rosengren, B. Järvholm and K. Torén (2015). "Psychosocial work environment and risk of ischemic stroke and coronary heart disease: a prospective longitudinal study of 75 236 construction workers." Scand J Work Environ Health **41**(3): 280-287.

Slopen, N., R. J. Glynn, J. E. Buring, T. T. Lewis, D. R. Williams and M. A. Albert (2012). "Job strain, job insecurity, and incident cardiovascular disease in the Women's Health Study: results from a 10-year prospective study." PLoS One **7**(7): e40512.

Smith, P., M. Gilbert-Ouimet, C. Brisson, R. H. Glazier and C. A. Mustard (2021). "Examining the relationship between the demand-control model and incident myocardial infarction and congestive heart failure in a representative sample of the employed women and men in Ontario, Canada, over a 15-year period." Can J Public Health **112**(2): 280-288.

Steenland, K., J. Johnson and S. Nowlin (1997). "A follow-up study of job strain and heart disease among males in the NHANES1 population." Am J Ind. Med **31**(2): 256-260.

Torén, K., L. Schiöler, W. K. Giang, M. Novak, M. Söderberg and A. Rosengren (2014). "A longitudinal general population-based study of job strain and risk for coronary heart disease and stroke in Swedish men." BMJ Open **4**(3): e004355.

Wang, C., F. Lê-Scherban, J. Taylor, E. Salmoirago-Blotcher, M. Allison, D. Gefen, L. Robinson and Y. L. Michael (2021). "Associations of Job Strain, Stressful Life Events, and Social Strain With Coronary Heart Disease in the Women's Health Initiative Observational Study." J Am Heart Assoc **10**(5): e017780.

IV Reasons for exclusion of full text read papers

Main grouping of ineligibility
1. **Not addressing DCS-model and/or ischemic heart disease**

Alicandro, G., P. Bertuccio, G. Sebastiani, C. La Vecchia and L. Frova (2020). "Long working hours and cardiovascular mortality: a census-based cohort study." Int J Public Health **65**(3): 257-266.

André-Petersson, L., G. Engström, B. Hedblad, L. Janzon and M. Rosvall (2007). "Social support at work and the risk of myocardial infarction and stroke in women and men." Soc Sci Med **64**(4): 830-841.

De Vogli, R., J. E. Ferrie, T. Chandola, M. Kivimäki and M. G. Marmot (2007). "Unfairness and health: evidence from the Whitehall II Study." J Epidemiol Community Health **61**(6): 513-518.

Dragano, N., J. Siegrist, S. Nyberg and M. Kivimaki (2017). "Effort-reward imbalance at work and job strain as risk factors for incident coronary heart disease: Results from the multicohort IPD-work consortium." European Journal of Preventive Cardiology **24**(2 Supplement 1): 11.

Ferrie, J. E., M. Kivimäki, M. J. Shipley, G. Davey Smith and M. Virtanen (2013). "Job insecurity and incident coronary heart disease: the Whitehall II prospective cohort study." Atherosclerosis **227**(1): 178-181.

Fransson, E. I., S. T. Nyberg, K. Heikkilä, L. Alfredsson, J. B. Bjorner, M. Borritz, H. Burr, N. Dragano, G. A. Geuskens, M. Goldberg, M. Hamer, W. E. Hooftman, I. L. Houtman, M. Joensuu, M. Jokela, A. Knutsson, M. Koskenvuo, A. Koskinen, M. Kumari, C. Leineweber, T. Lunau, I. E. Madsen, L. L. Hanson, M. L. Nielsen, M. Nordin, T. Oksanen, J. Pentti, J. H. Pejtersen, R. Rugulies, P. Salo, M. J. Shipley, A. Steptoe, S. B. Suominen, T. Theorell, S. Toppinen-Tanner, J. Vahtera, M. Virtanen, A. Väänänen, P. J. Westerholm, H. Westerlund, M. Zins, A. Britton, E. J. Brunner, A. Singh-Manoux, G. D. Batty and M. Kivimäki (2015). "Job strain and the risk of stroke: an individual-participant data meta-analysis." Stroke **46**(2): 557-559.

Hannerz, H., K. Albertsen, H. Burr, M. L. Nielsen, A. H. Garde, A. D. Larsen and J. H. Pejtersen (2018). "Long working hours and stroke among employees in the general workforce of Denmark." Scand J Public Health **46**(3): 368-374.

Hannerz, H., A. D. Larsen and A. H. Garde (2018). "Long weekly working hours and ischaemic heart disease: a follow-up study among 145 861 randomly selected workers in Denmark." BMJ Open **8**(6): e019807.

Hayashi, R., H. Iso, K. Yamagishi, H. Yatsuya, I. Saito, Y. Kokubo, E. S. Eshak, N. Sawada and S. Tsugane (2019). "Working Hours and Risk of Acute Myocardial Infarction and Stroke Among Middle-Aged Japanese Men - The Japan Public Health Center-Based Prospective Study Cohort II." Circ J **83**(5): 1072-1079.

Heslop, P., G. D. Smith, C. Metcalfe, J. Macleod and C. Hart (2002). "Change in job satisfaction, and its association with self-reported stress, cardiovascular risk factors and mortality." Soc Sci Med **54**(10): 1589-1599.

Hibbard, J. H. and C. R. Pope (1993). "The quality of social roles as predictors of morbidity and mortality." Soc Sci Med **36**(3): 217-225.

Holtermann, A., O. S. Mortensen, H. Burr, K. Søgaard, F. Gyntelberg and P. Suadicani (2010). "Long work hours and physical fitness: 30-year risk of ischaemic heart disease and all-cause mortality among middle-aged Caucasian men." Heart **96**(20): 1638-1644.

Huisman, M., F. Van Lenthe, M. Avendano and J. Mackenbach (2008). "The contribution of job characteristics to socioeconomic inequalities in incidence of myocardial infarction." Soc Sci Med **66**(11): 2240-2252.

Jensen, J. H., E. M. Flachs, J. Skakon, N. H. Rod, J. P. Bonde and I. Kawachi (2020). "Work-unit organizational changes and risk of cardiovascular disease: a prospective study of public healthcare employees in Denmark." Int Arch Occup Environ Health **93**(4): 409-419.

Kc, P., I. E. H. Madsen, R. Rugulies, T. Xu, H. Westerlund, A. Nyberg, M. Kivimäki and L. L. M. Hanson (2024). "Exposure to workplace sexual harassment and risk of cardiometabolic disease: a prospective cohort study of 88 904 Swedish men and women." Eur J Prev Cardiol **31**(13): 1633-1642.

Kivimäki, M., J. E. Ferrie, E. Brunner, J. Head, M. J. Shipley, J. Vahtera and M. G. Marmot (2005). "Justice at work and reduced risk of coronary heart disease among employees: the Whitehall II Study." Arch Intern Med **165**(19): 2245-2251.

Kivimäki, M., J. E. Ferrie, M. Shipley, D. Gimeno, M. Elovainio, R. de Vogli, J. Vahtera, M. G. Marmot and J. Head (2008). "Effects on blood pressure do not explain the association between organizational justice and coronary heart disease in the Whitehall II study." Psychosom Med **70**(1): 1-6.

Kivimäki, M., D. Gimeno, J. E. Ferrie, G. D. Batty, T. Oksanen, M. Jokela, M. Virtanen, P. Salo, T. N. Akbaraly, M. Elovainio, J. Pentti and J. Vahtera (2009). "Socioeconomic position, psychosocial work environment and cerebrovascular disease among women: the Finnish public sector study." Int J Epidemiol **38**(5): 1265-1271.

Kivimäki, M., M. Jokela, S. T. Nyberg, A. Singh-Manoux, E. I. Fransson, L. Alfredsson, J. B. Bjorner, M. Borritz, H. Burr, A. Casini, E. Clays, D. De Bacquer, N. Dragano, R. Erbel, G. A. Geuskens, M. Hamer, W. E. Hooftman, I. L. Houtman, K. H. Jöckel, F. Kittel, A. Knutsson, M. Koskenvuo, T.

Lunau, I. E. Madsen, M. L. Nielsen, M. Nordin, T. Oksanen, J. H. Pejtersen, J. Pentti, R. Rugulies, P. Salo, M. J. Shipley, J. Siegrist, A. Steptoe, S. B. Suominen, T. Theorell, J. Vahtera, P. J. Westerholm, H. Westerlund, D. O'Reilly, M. Kumari, G. D. Batty, J. E. Ferrie and M. Virtanen (2015). "Long working hours and risk of coronary heart disease and stroke: a systematic review and meta-analysis of published and unpublished data for 603,838 individuals." Lancet **386**(10005): 1739-1746.

Kivimäki, M., P. Leino-Arjas, R. Luukkonen, H. Riihimäki, J. Vahtera and J. Kirjonen (2002). "Work stress and risk of cardiovascular mortality: prospective cohort study of industrial employees." Bmj **325**(7369): 857.

Kivimäki, M., T. Theorell, H. Westerlund, J. Vahtera and L. Alfredsson (2008). "Job strain and ischaemic disease: does the inclusion of older employees in the cohort dilute the association? The WOLF Stockholm Study." J Epidemiol Community Health **62**(4): 372-374.

Kornitzer, M., P. deSmet, S. Sans, M. Dramaix, C. Boulenguez, G. DeBacker, M. Ferrario, I. Houtman, S. O. Isacsson, P. O. Ostergren, I. Peres, E. Pelfrene, M. Romon, A. Rosengren, G. Cesana and L. Wilhelmsen (2006). "Job stress and major coronary events: results from the Job Stress, Absenteeism and Coronary Heart Disease in Europe study." Eur J Cardiovasc Prev Rehabil **13**(5): 695-704.

Kuper, H., H. O. Adami, T. Theorell and E. Weiderpass (2007). "The socioeconomic gradient in the incidence of stroke: a prospective study in middle-aged women in Sweden." Stroke **38**(1): 27-33.

Kuper, H., A. Singh-Manoux, J. Siegrist and M. Marmot (2002). "When reciprocity fails: effort-reward imbalance in relation to coronary heart disease and health functioning within the Whitehall II study." Occup Environ Med **59**(11): 777-784.

Latza, U., K. Rossnagel, H. Hannerz, H. Burr, S. Jankowiak and E. M. Backé (2015). "Association of perceived job insecurity with ischemic heart disease and antihypertensive medication in the Danish Work Environment Cohort Study 1990-2010." Int Arch Occup Environ Health **88**(8): 1087-1097.

Lee, S., G. A. Colditz, L. F. Berkman and I. Kawachi (2004). "Prospective study of job insecurity and coronary heart disease in US women." Ann Epidemiol **14**(1): 24-30.

Leineweber, C., H. Westerlund, T. Theorell, M. Kivimäki, P. Westerholm and L. Alfredsson (2011). "Covert coping with unfair treatment at work and risk of incident myocardial infarction and cardiac death among men: prospective cohort study." J Epidemiol Community Health **65**(5): 420-425.

Lynch, J., N. Krause, G. A. Kaplan, J. Tuomilehto and J. T. Salonen (1997). "Workplace conditions, socioeconomic status, and the risk of mortality and acute myocardial infarction: the Kuopio Ischemic Heart Disease Risk Factor Study." Am J Public Health **87**(4): 617-622.

Martikainen, P., N. Mäki and M. Jäntti (2008). "The effects of workplace downsizing on cause-specific mortality: a register-based follow-up study of Finnish men and women remaining in employment." J Epidemiol Community Health **62**(11): 1008-1013.

O'Reilly, D. and M. Rosato (2013). "Worked to death? A census-based longitudinal study of the relationship between the numbers of hours spent working and mortality risk." Int J Epidemiol **42**(6): 1820-1830.

Padyab, M., Y. Blomstedt and M. Norberg (2014). "No association found between cardiovascular mortality, and job demands and decision latitude: experience from the Västerbotten Intervention Programme in Sweden." Soc Sci Med **117**: 58-66.

Sokejima, S. and S. Kagamimori (1998). "Working hours as a risk factor for acute myocardial infarction in Japan: case-control study." Bmj **317**(7161): 775-780.

Theorell, T. and B. Floderus-Myrhed (1977). "'Workload' and risk of myocardial infarction--a prospective psychosocial analysis." Int J Epidemiol **6**(1): 17-21.

Tsutsumi, A., K. Kayaba and S. Ishikawa (2011). "Impact of occupational stress on stroke across occupational classes and genders." Soc Sci Med **72**(10): 1652-1658.

Vahtera, J., M. Kivimäki, J. Pentti, A. Linna, M. Virtanen, P. Virtanen and J. E. Ferrie (2004). "Organisational downsizing, sickness absence, and mortality: 10-town prospective cohort study." Bmj **328**(7439): 555.

Virtanen, M., J. E. Ferrie, A. Singh-Manoux, M. J. Shipley, J. Vahtera, M. G. Marmot and M. Kivimäki (2010). "Overtime work and incident coronary heart disease: the Whitehall II prospective cohort study." Eur Heart J **31**(14): 1737-1744.

Virtanen, S. V. and V. Notkola (2002). "Socioeconomic inequalities in cardiovascular mortality and the role of work: a register study of Finnish men." Int J Epidemiol **31**(3): 614-621.

Väänänen, A., A. Koskinen, M. Joensuu, M. Kivimäki, J. Vahtera, A. Kouvonen and P. Jäppinen (2008). "Lack of predictability at work and risk of acute myocardial infarction: an 18-year prospective study of industrial employees." Am J Public Health **98**(12): 2264-2271.

Wu, W. T., S. S. Tsai, C. C. Wang, Y. J. Lin, T. N. Wu, T. S. Shih and S. H. Liou (2019). "Professional Driver's Job Stress and 8-year Risk of Cardiovascular Disease: The Taiwan Bus Driver Cohort Study." Epidemiology **30 Suppl 1**: S39-s47.

Xu, T., L. L. Magnusson Hanson, T. Lange, L. Starkopf, H. Westerlund, I. E. H. Madsen, R. Rugulies, J. Pentti, S. Stenholm, J. Vahtera, M. Hansen Å, M. Virtanen, M. Kivimäki and N. H. Rod (2019). "Workplace bullying and workplace violence as risk factors for cardiovascular disease: a multi-cohort study." Eur Heart J **40**(14): 1124-1134.

Xu, T., R. Rugulies, J. Vahtera, J. Pentti, J. Mathisen, T. Lange, A. J. Clark, L. L. Magnusson Hanson, H. Westerlund, J. Ervasti, M. Virtanen, M. Kivimäki and N. H. Rod (2022). "Workplace psychosocial resources and risk of cardiovascular disease among employees: a multi-cohort study of 135 669 participants." Scand J Work Environ Health **48**(8): 621-631.

**2. Design issues**		**Specific reasons for ineligibility**
	Karasek, R. A., T. G. Theorell, J. Schwartz, C. Pieper and L. Alfredsson (1982). "Job, psychological factors and coronary heart disease. Swedish prospective findings and US prevalence findings using a new occupational inference method." Adv Cardiol **29**: 62-67.	Some estimates are cross-sectional. Other estimates from case-control studies not adjusted for socio-economic class.
	Alfredsson, L., C. L. Spetz and T. Theorell (1985). "Type of occupation and near-future hospitalization for myocardial infarction and some other diagnoses." Int. J Epidemiol 14(3): 378-388.	Selective reporting of significant associations only between a large number of JEM based work characteristics and IHD and stroke.
	Bobák, M., C. Hertzman, Z. Skodová and M. Marmot (1998). "Association between psychosocial factors at work and nonfatal myocardial infarction in a population-based case-control study in Czech men." Epidemiology 9(1): 43-47.	Hospital-based case control study with retrospective questionnaire information and IHD diagnoses including non-definite cases.
	Hallqvist, J., F. Diderichsen, T. Theorell, C. Reuterwall and A. Ahlbom (1998). "Is the effect of job strain on myocardial infarction risk due to interaction between high psychological demands and low decision latitude? Results from Stockholm Heart Epidemiology Program (SHEEP)." Soc Sci Med 46(11): 1405-1415	Hospital-based case control study with retrospective questionnaire information.
	Herbert, R., C. Schechter, D. A. Smith, R. Phillips, J. Diamond, S. Carroll, J. Weiner, T. E. Dahms and P. J. Landrigan (2000). "Occupational coronary heart disease among bridge and tunnel officers." Arch Environ Health 55(3): 152-163.	Cross-sectional study using prevalence data.
	Reuterwall, C., J. Hallqvist, A. Ahlbom, U. De Faire, F. Diderichsen, C. Hogstedt, G. Pershagen, T. Theorell, B. Wiman and A. Wolk (1999). "Higher relative, but lower absolute risks of myocardial infarction in women than in men: analysis of some major risk factors in the SHEEP study. The SHEEP Study Group." J Intern Med 246(2): 161-174	Case control study (SHEEP) with retrospective questionnaire information on psychosocial and other exposures.
	Orth-Gomér, K., S. P. Wamala, M. Horsten, K. Schenck-Gustafsson, N. Schneiderman and M. A. Mittleman (2000). "Marital stress worsens prognosis in women with coronary heart disease: The Stockholm Female Coronary Risk Study." Jama 284(23): 3008-3014.	Risk of recurrent CHD in women hospitalized for acute AMI or unstable angina pectoris
	Wamala, S. P., M. A. Mittleman, M. Horsten, K. Schenck-Gustafsson and K. Orth-Gomér (2000). "Job stress and the occupational gradient in coronary heart disease risk in women. The Stockholm Female Coronary Risk Study." Soc Sci Med 51(4): 481-489.	Case control study with retrospective questionnaire information on psychosocial and other exposures.
	Sacker, A., M. J. Bartley, D. Frith, R. M. Fitzpatrick and M. G. Marmot (2001). "The relationship between job strain and coronary heart disease: evidence from an english sample of the working male population." Psychol Med 31(2): 279-290.	Cross-sectional survey addressing job strain using self-reported physician diagnosed IHD.
	Uchiyama, S., T. Kurasawa, T. Sekizawa and H. Nakatsuka (2005). "Job strain and risk of cardiovascular events in treated hypertensive Japanese workers: hypertension follow-up group study." J Occup. Health 47(2): 102-111.	Study population selected by CVD status (treated hypertension).
	Kopp, M., A. Skrabski, Z. Szántó and J. Siegrist (2006). "Psychosocial determinants of premature cardiovascular mortality differences within Hungary." J Epidemiol Community Health 60(9): 782-788.	Cross-sectional ecologic study design.
	Demiral, Y., A. Soysal, A. Can Bilgin, B. Kiliç, B. Unal, R. Uçku and T. Theorell (2006). "The association of job strain with coronary heart disease and metabolic syndrome in municipal workers in Turkey." J Occup Health 48(5): 332-338.	Cross-sectional study.
	Mc Carthy, V. J., I. J. Perry and B. A. Greiner (2012). "Age, job characteristics and coronary health." Occup Med (Lond) 62(8): 613-619.	Case-control study with self-reported exposure data.
	Selander, J., G. Bluhm, M. Nilsson, J. Hallqvist, T. Theorell, P. Willix and G. Pershagen (2013). "Joint effects of job strain and road-traffic and occupational noise on myocardial infarction." Scand J Work Environ Health 39(2): 195-203	Case control study (SHEEP) with retrospective questionnaire information on psychosocial and other exposures.
	Cheng, Y., C. L. Du, J. J. Hwang, I. S. Chen, M. F. Chen and T. C. Su (2014). "Working hours, sleep duration and the risk of acute coronary heart disease: a case-control study of middle-aged men in Taiwan." Int J Cardiol 171(3): 419-422.	Case-control study with retrospective self-reported exposure (average working hours the week before hospital admission for CHD)
	Kang, M. Y., S. H. Cho, M. S. Yoo, T. Kim and Y. C. Hong (2014). "Long working hours may increase risk of coronary heart disease." Am J Ind Med 57(11): 1227-1234.	Cross-sectional study design.

	Becher, H., M. F. Dollard, P. Smith and J. Li (2018). "Predicting Circulatory Diseases from Psychosocial Safety Climate: A Prospective Cohort Study from Australia." Int J Environ Res Public Health 15(3).	Self-reported cardiovascular disease: 'In the past two years have you consulted a health professional regarding chest pain, or any other cardiovascular related health problem—such as myocardial infarction, angina, stroke or hypertension?'
	Chen, W. L., C. C. Wang, S. T. Chiang, Y. C. Wang, Y. S. Sun, W. T. Wu and S. H. Liou (2018). "The impact of occupational psychological hazards and metabolic syndrome on the 8-year risk of cardiovascular diseases-A longitudinal study." PLoS One **13**(8): e0202977.	Outcome and exposure ascertainment insufficiently described. The ICD-10 diagnoses include numerous diagnoses not mentioned. The incidence of CVD during 7 years among men 43 years at entry of 155/10,000 is very high and unexplained. Outcome data partly form database (long working hours), but no details are given. Jobstrain not defined.
	Fadel, M., G. Sembajwe, D. Gagliardi, F. Pico, J. Li, A. Ozguler, J. Siegrist, B. A. Evanoff, M. Baer, A. Tsutsumi, S. Iavicoli, A. Leclerc, Y. Roquelaure and A. Descatha (2019). "Association Between Reported Long Working Hours and History of Stroke in the CONSTANCES Cohort." Stroke **50**(7): 1879-1882.	Cross-sectional study with self-reported stroke outcome (reported to physician at interview).
	Magnusson Hanson, L. L., N. H. Rod, J. Vahtera, P. Peristera, J. Pentti, R. Rugulies, I. E. H. Madsen, A. D. LaMontagne, A. Milner, T. Lange, S. Suominen, S. Stenholm, T. Xu, M. Kivimäki and H. Westerlund (2019). "Multicohort study of change in job strain, poor mental health and incident cardiometabolic disease." Occup Environ Med **76**(11): 785-792.	The outcome includes diabetes (cardiometabolic syndrome) and is partly self-reported (in one of 5 cohorts)
Exposure issues		
	Haynes SG, Feinleib M, Kannel WB. The relationship of psychosocial factors to coronary heart disease in the Framingham Study. III. Eight-year incidence of coronary heart disease. Am J Epidemiol. 1980;111(1):37-58.	No data on effect of work-related psychosocial exposures.
	Tüchsen, F. (1993). "Working hours and ischaemic heart disease in Danish men: a 4-year cohort study of hospitalization." Int J Epidemiol **22**(2): 215-221.	Addresses night and shift work, not long working hours or other workplace psychosocial exposures
	Matthews, K. A. and B. B. Gump (2002). "Chronic work stress and marital dissolution increase risk of posttrial mortality in men from the Multiple Risk Factor Intervention Trial." Arch Intern Med **162**(3): 309-315.	Works stress includes a broad range of difficulties related to work (e.g. being fired) and estimates only provided for a number of exposures without specifying which ones. The study population is men with above average risk of CVD as judged by baseline presence of CVD risk factors
	De Vogli, R., J. E. Ferrie, T. Chandola, M. Kivimaki and M. G. Marmot (2007). "Unfairness and health: evidence from the Whitehall II Study." J Epidemiol. Community Health **61**(6): 513-518.	Exposure is unfairness but includes unfair treatment in general and not limited to the workplace, which the authors stress explicitly.
	Macleod, J., C. Metcalfe, G. D. Smith and C. Hart (2007). "Does consideration of either psychological or material disadvantage improve coronary risk prediction? Prospective observational study of Scottish men." J Epidemiol Community Health **61**(9): 833-837.	No data on effects of explicit work-related psychosocial exposures.
	Nabi, H., M. Kivimaki, R. De Vogli, M. G. Marmot and A. Singh-Manoux (2008). "Positive and negative affect and risk of coronary heart disease: Whitehall II prospective cohort study." Bmj **337**(7660): a118	No analyses on effect of work-related psychosocial exposures.
	Leineweber C, Westerlund H, Theorell T, Kivimäki M, Westerholm P, Alfredsson L. Covert coping with unfair treatment at work and risk of incident myocardial infarction and cardiac death among men: prospective cohort study. J Epidemiol Community Health. 2011;65(5):420-5	No explicit occupational exposure. Concerns how an individual react to unfairness (coping rather than exposure).
	Kersten, N. and E. Backé (2015). "Occupational noise and myocardial infarction: considerations on the interrelation of noise with job demands." Noise Health **17**(75): 116-122.	No data on effect of work-related psychosocial exposures.
	Schnohr, P., J. L. Marott, T. S. Kristensen, F. Gyntelberg, M. Grønbæk, P. Lange, M. T. Jensen, G. B. Jensen and E. Prescott (2015). "Ranking of psychosocial and traditional risk factors by importance for coronary heart disease: the Copenhagen City Heart Study." Eur Heart J **36**(22): 1385-1393	Risk estimates not adjusted for sex and SES.
	Tillmann, T., H. Pikhart, A. Peasey, R. Kubinova, A. Pajak, A. Tamosiunas, S. Malyutina, A. Steptoe, M. Kivimäki, M. Marmot and M. Bobak (2017). "Psychosocial and socioeconomic determinants of cardiovascular mortality in Eastern Europe: A multicentre prospective cohort study." PLoS Med **14**(12): e1002459.	No data on effect of work-related psychosocial exposures. Pooling of CVD mortality outcomes (ICD-10 I00-I99).
	Cohen, H. W., R. Zeig-Owens, C. Joe, C. B. Hall, M. P. Webber, M. D. Weiden, K. L. Cleven, N. Jaber, M. Skerker, J. Yip, T. Schwartz and D. J. Prezant (2019). "Long-term Cardiovascular Disease Risk Among Firefighters After the World Trade Center Disaster." JAMA Netw Open **2**(9): e199775	No data on effects of explicit work-related psychosocial exposures. Composite outcome (pooling of CVD diagnostic subtypes, e.g. AMI and stroke).
	Walli-Attaei, M., A. Rosengren, S. Rangarajan, Y. Breet, S. Abdul-Razak, W. A. Sharief, K. F. Alhabib, A. Avezum, J. Chifamba, R. Diaz, R. Gupta, B. Hu, R. Iqbal, R. Ismail, R. Kelishadi, R. Khatib, X. Lang, S. Li, P. Lopez-Jaramillo, V. Mohan, A. Oguz, L. M. Palileo-Villanueva, K. Poltyn-Zaradna, S. P. R, L. V. M. Pinnaka, P. Serón, K. Teo, S. T. Verghese, A. Wielgosz, K. Yeates, R. Yusuf, S. S. Anand and S. Yusuf (2022). "Metabolic, behavioural, and psychosocial risk factors and cardiovascular disease in women compared with men in 21	No data on effect of work-related psychosocial exposures.

	high-income, middle-income, and low-income countries: an analysis of the PURE study." Lancet **400**(10355): 811-821.	
	Santosa, A., A. Rosengren, C. Ramasundarahettige, S. Rangarajan, S. Gulec, J. Chifamba, S. A. Lear, P. Poirier, K. E. Yeates, R. Yusuf, A. Orlandini, L. Weida, L. Sidong, Z. Yibing, V. Mohan, M. Kaur, K. Zatonska, N. Ismail, P. Lopez-Jaramillo, R. Iqbal, L. M. Palileo-Villanueva, A. H. Yusufali, K. F. AlHabib and S. Yusuf (2021). "Psychosocial Risk Factors and Cardiovascular Disease and Death in a Population-Based Cohort From 21 Low-, Middle-, and High-Income Countries." JAMA Netw Open **4**(12): e2138920.	Pools work and non-work questions on stress reactions rather than exposure to psychosocial stressors.
	Ajibewa, T. A., K. N. Kershaw, J. J. Carr, J. G. Terry, K. P. Gabriel, M. R. Carnethon, M. Wong and N. B. Allen (2024). "Chronic Stress and Cardiovascular Events: Findings From the CARDIA Study." Am J Prev Med **67**(1): 24-31.	Applies a stress score from 5 domains not distinguishing work related stressors.
Outcome issues		
	Johnson, J. V., E. M. Hall and T. Theorell (1989). "Combined effects of job strain and social isolation on cardiovascular disease morbidity and mortality in a random sample of the Swedish male working population." Scand J Work Environ Health **15**(4): 271-279.	Addresses various cardiovascular disorders assembled in one variable: hypertension, ischemic heart disease, cerebrovascular disease and more.
	Siegrist, J., R. Peter, A. Junge, P. Cremer and D. Seidel (1990). "Low status control, high effort at work and ischemic heart disease: prospective evidence from blue-collar men." Soc Sci Med **31**(10): 1127-1134.	Outcomes include probable coronary heart disease in addition to definite cases.
	Siegrist, J., R. Peter, W. Motz and B. E. Strauer (1992). "The role of hypertension, left ventricular hypertrophy and psychosocial risks in cardiovascular disease: prospective evidence from blue-collar men." Eur Heart J **13 Suppl D**: 89-95.	Outcomes include probable coronary heart disease in addition to definite cases.
	Siegrist, J. and R. Peter (1994). "Job stressors and coping characteristics in work-related disease: Issues of validity." Work and Stress **8**(2): 130 EP - 140.	Broadly defined CHD outcome (indefinite or probable CHD).
	Johnson, J. V., W. Stewart, E. M. Hall, P. Fredlund and T. Theorell (1996). "Long-term psychosocial work environment and cardiovascular mortality among Swedish men." Am J Public Health **86**(3): 324-331.	Addresses various cardiovascular disorders assembled in one variable: ischemic heart disease, cerebrovascular disease and peripheral arterial disease.
	Marmot, M. G., H. Bosma, H. Hemingway, E. Brunner and S. Stansfeld (1997). "Contribution of job control and other risk factors to social variations in coronary heart disease incidence." Lancet **350**(9073): 235-239.	Self-reported CHD outcomes
	Bosma, H., R. Peter, J. Siegrist and M. Marmot (1998). "Two alternative job stress models and the risk of coronary heart disease." Am J Public Health **88**(1): 68-74.	Outcome defined by self-reported angina pectoris or self-reported doctor diagnosed ischemic heart disease.
	Amick, B. C., 3rd, P. McDonough, H. Chang, W. H. Rogers, C. F. Pieper and G. Duncan (2002). "Relationship between all-cause mortality and cumulative working life course psychosocial and physical exposures in the United States labor market from 1968 to 1992." Psychosom Med **64**(3): 370-381.	Addresses all-cause mortality**. N**o data on IHD or stroke mortality.
	Kivimäki, M., P. Leino-Arjas, R. Luukkonen, H. Riihimäki, J. Vahtera and J. Kirjonen (2002). "Work stress and risk of cardiovascular mortality: prospective cohort study of industrial employees." Bmj **325**(7369): 857.	CVD mortality outcome includes a range of CVDs in addition to IHD and stroke
	Kivimäki, M., M. Virtanen, M. Vartia, M. Elovainio, J. Vahtera and L. Keltikangas-Järvinen (2003). "Workplace bullying and the risk of cardiovascular disease and depression." Occup Environ Med **60**(10): 779-783.	Uses self-reported CHD data.
	Elovainio, M., P. Leino-Arjas, J. Vahtera and M. Kivimäki (2006). "Justice at work and cardiovascular mortality: a prospective cohort study." J Psychosom Res **61**(2): 271-274	Outcome is CVD mortality including the entire chapter of CVD.
	Toivanen, S. and O. Hemström (2006). "Income differences in cardiovascular disease: is the contribution from work similar in prevalence versus mortality outcomes?" Int J Behav Med **13**(1): 89-100	Outcome is CVD mortality including pulmonary and peripheral vascular diseases.
	Oksanen, T., M. Kivimäki, I. Kawachi, S. V. Subramanian, S. Takao, E. Suzuki, A. Kouvonen, J. Pentti, P. Salo, M. Virtanen and J. Vahtera (2011). "Workplace social capital and all-cause mortality: a prospective cohort study of 28,043 public-sector employees in Finland." Am J Public Health **101**(9): 1742-1748	Addresses all-cause mortality**,** no data on IHD or stroke mortality.
	Toker, S., S. Melamed, S. Berliner, D. Zeltser and I. Shapira (2012). "Burnout and risk of coronary heart disease: a prospective study of 8838 employees." Psychosom Med **74**(8): 840-847.	Self-reported IHD outcomes only partially medically verified.
	Szerencsi, K., L. van Amelsvoort, J. Serroyen, M. Prins, N. Jansen and I. Kant (2013). "The impact of personal attributes on the association between cumulative exposure to work stressors and cardiovascular disease." J Psychosom Res **75**(1): 23-31	Addresses cardiovascular disease without separate analyses of ischemic heart disease or stroke. Self-reported outcome (but medically verified in half of the cohort).
	Nilsen, C., R. Andel, J. Fritzell and I. Kåreholt (2016). "Work-related stress in midlife and all-cause mortality: can sense of coherence modify this association?" Eur J Public Health **26**(6): 1055-1061.	Addresses all-cause mortality without data on IHD or stroke mortality.
	Marchand, A., M. E. Blanc and N. Beauregard (2017). "Exposure to Work and Nonwork Stressors and the Development of Heart Disease Among	Self-reported heart disease outcome.

	Canadian Workers Aged 40 Years and Older: A 16-year Follow-up Study (1994 to 2010)." J Occup Environ Med **59**(9): 894-902.	
	Shin, K. S., Y. K. Chung, Y. J. Kwon, J. S. Son and S. H. Lee (2017). "The effect of long working hours on cerebrovascular and cardiovascular disease; A case-crossover study." Am J Ind Med **60**(9): 753-761	Case-cross over study with retrospective self-reported exposure (average weekly working hours) during specified time-periods before hospitalisation.
	Kivimäki, M., J. Pentti, J. E. Ferrie, G. D. Batty, S. T. Nyberg, M. Jokela, M. Virtanen, L. Alfredsson, N. Dragano, E. I. Fransson, M. Goldberg, A. Knutsson, M. Koskenvuo, A. Koskinen, A. Kouvonen, R. Luukkonen, T. Oksanen, R. Rugulies, J. Siegrist, A. Singh-Manoux, S. Suominen, T. Theorell, A. Väänänen, J. Vahtera, P. J. M. Westerholm, H. Westerlund, M. Zins, T. Strandberg, A. Steptoe and J. Deanfield (2018). "Work stress and risk of death in men and women with and without cardiometabolic disease: a multicohort study." Lancet Diabetes Endocrinol **6**(9): 705-713.	Addresses all-cause mortality without separate analyses of ischemic heart disease and stroke.
	Hayashi, R., H. Iso, K. Yamagishi, H. Yatsuya, I. Saito, Y. Kokubo, E. S. Eshak, N. Sawada and S. Tsugane (2019). "Working Hours and Risk of Acute Myocardial Infarction and Stroke Among Middle-Aged Japanese Men - The Japan Public Health Center-Based Prospective Study Cohort II." Circ J 83(5): 1072-1079.	IHD outcome include possible, not medically verified IHD. (However, the study also reports risk of stroke which is not excluded).
	Heslop, P., G. D. Smith, C. Metcalfe, J. Macleod and C. Hart (2002). "Change in job satisfaction, and its association with self-reported stress, cardiovascular risk factors and mortality." Soc Sci Med **54**(10): 1589-1599.	CVD mortality broadly defined (ICD9 390-459) including other CVD than IHD and stroke.
	Lee, W., Y. J. Kang, T. Kim, J. Choi and M. Y. Kang (2019). "The Impact of Working Hours on Cardiovascular Diseases and Moderating Effects of Sex and Type of Work: Results From a Longitudinal Analysis of the Korean Working Population." J Occup Environ Med **61**(6): e247-e252.	Outcome (CVD) defined by questionnaire self-report without clinical verification.
	Romero Starke K, Hegewald J, Schulz A, Garthus-Niegel S, Nubling M, Wild PS, et al. Cardiovascular health outcomes of mobbing at work: Results of the population-based, five-year follow-up of the Gutenberg health study. Journal of Occupational Medicine and Toxicology. 2020;15(1):15.	Addresses cardiovascular disease without separate analyses of ischemic heart disease or stroke. Peripheral disorders are also included in the outcome.
	Sørensen, J. K., E. Framke, J. Pedersen, K. Alexanderson, J. P. Bonde, K. Farrants, E. M. Flachs, L. L. Magnusson Hanson, S. T. Nyberg, M. Kivimäki, I. E. H. Madsen and R. Rugulies (2022). "Work stress and loss of years lived without chronic disease: an 18-year follow-up of 1.5 million employees in Denmark." Eur J Epidemiol.	Outcome 'chronic disease' including a range of diseases in addition to CVD.
**3. Analysis issues**		**Specific reasons for ineligibility**
	Alfredsson, L., R. Karasek and T. Theorell (1982). "Myocardial infarction risk and psychosocial work environment: an analysis of the male Swedish working force." Soc Sci Med **16**(4): 463-467.	Risk estimates not adjusted for socio-economic position. Case-control study partly with self-reports on psychosocial exposures.
	Alfredsson, L. and T. Theorell (1983). "Job characteristics of occupations and myocardial infarction risk:effect of possible confounding factors." Soc Sci Med **17**(20): 1497-1503.	Case-control study partly with self-reports on psychosocial exposures.
	Reed, D. M., A. Z. LaCroix, R. A. Karasek, D. Miller and C. A. MacLean (1989). "Occupational strain and the incidence of coronary heart disease." Am J Epidemiol **129**(3): 495-502.	No risk estimates – only age-adjusted CHD incidence rates in male employees in Hawaii. No adjustment for socioeconomic class.
	Suadicani, P., H. O. Hein and F. Gyntelberg (1993). "Are social inequalities as associated with the risk of ischaemic heart disease a result of psychosocial working conditions?" Atherosclerosis **101**(2): 165-175.	No reporting of adjusted risk estimates.
	Andersen, I., H. Burr, T. S. Kristensen, M. Gamborg, M. Osler, E. Prescott and F. Diderichsen (2004). "Do factors in the psychosocial work environment mediate the effect of socioeconomic position on the risk of myocardial infarction? Study from the Copenhagen Centre for Prospective Population Studies." Occup Environ Med **61**(11): 886-892	No adjusted estimates of associations between JEM-based psychosocial exposures and risk of AMI.
	Tenkanen, L., T. Sjöblom, R. Kalimo, T. Alikoski and M. Härmä (1997). "Shift work, occupation and coronary heart disease over 6 years of follow-up in the Helsinki Heart Study." Scand J Work Environ Health **23**(4): 257-265.	No risk estimates for work-related psychosocial factors.
	Kornitzer, M., P. deSmet, S. Sans, M. Dramaix, C. Boulenguez, G. DeBacker, M. Ferrario, I. Houtman, S. O. Isacsson, P. O. Ostergren, I. Peres, E. Pelfrene, M. Romon, A. Rosengren, G. Cesana and L. Wilhelmsen (2006). "Job stress and major coronary events: results from the Job Stress, Absenteeism and Coronary Heart Disease in Europe study." Eur. J Cardiovasc. Prev. Rehabil **13**(5): 695-704.	Risk estimates not controlled for SES.
	Netterstrøm, B., T. S. Kristensen and A. Sjøl (2006). "Psychological job demands increase the risk of ischaemic heart disease: a 14-year cohort study of employed Danish men." Eur J Cardiovasc Prev Rehabil **13**(3): 414-420.	Insufficient reporting of data analysis. Statistical model with too few details to allow interpretation of provided risk estimates. Analyses by continuous exposure sum scores or dichotomous outcome. Job strain variable in 4 categories but split values not provided.
	Kivimaki, M., D. Gimeno, J. E. Ferrie, G. D. Batty, T. Oksanen, M. Jokela, M. Virtanen, P. SalO, T. N. Akbaraly, M. Elovainio, J. Pentti and J. Vahtera (2009). "Socioeconomic position, psychosocial work environment and cerebrovascular disease among women: the Finnish public sector study." Int. J. Epidemiol **38**(5): 1265-1271.	Risk estimates not adjusted for SES.

	Ferrario, M. M., G. Veronesi, L. E. Chambless, R. Sega, C. Fornari, M. Bonzini and G. Cesana (2011). "The contribution of major risk factors and job strain to occupational class differences in coronary heart disease incidence: the MONICA Brianza and PAMELA population-based cohorts." Occup Environ Med **68**(10): 717-722.	Risk estimates for psychosocial exposures are not provided (only adjustment by job strain).
	Emeny, R. T., A. Zierer, M. E. Lacruz, J. Baumert, C. Herder, G. Gornitzka, W. Koenig, B. Thorand and K. H. Ladwig (2013). "Job strain-associated inflammatory burden and long-term risk of coronary events: findings from the MONICA/KORA Augsburg case-cohort study." Psychosom Med **75**(3): 317-325.	Case-control study not adjusting for socio-economic position.
	Eriksson, H. P., E. Andersson, L. Schiöler, M. Söderberg, M. Sjöström, A. Rosengren and K. Torén (2018). "Longitudinal study of occupational noise exposure and joint effects with job strain and risk for coronary heart disease and stroke in Swedish men." BMJ Open **8**(4): e019160	Risk estimates for psychosocial exposures are not provided.
	Framke, E., J. K. Sørensen, P. K. Andersen, A. C. Svane-Petersen, K. Alexanderson, J. P. Bonde, K. Farrants, E. M. Flachs, L. L. M. Hanson, S. T. Nyberg, E. Villadsen, M. Kivimäki, R. Rugulies and I. E. H. Madsen (2020). "Contribution of income and job strain to the association between education and cardiovascular disease in 1.6 million Danish employees." Eur Heart J **41**(11): 1164-1178	Risk estimates for psychosocial exposures are not provided.
	Lee, W., J. Lee, H. R. Kim, Y. M. Lee, D. W. Lee and M. Y. Kang (2021). "The combined effect of long working hours and individual risk factors on cardiovascular disease: An interaction analysis." J Occup Health **63**(1): e12204.	Relevant risk estimates for long working hours are not provided.
	Almroth, M., T. Hemmingsson, D. Falkstedt, K. Kjellberg, E. Carlsson, K. Y. Pan, K. Berglund and E. Thern (2024). "The role of working conditions in educational differences in all-cause and ischemic heart disease mortality among Swedish men." Scand J Work Environ Health **50**(4): 300-309	Risk estimates for psychosocial exposures are not provided.
**4. Overlapping data**		**Specific reasons for ineligibility**
	Hammar, N., L. Alfredsson and T. Theorell (1994). "Job characteristics and the incidence of myocardial infarction." Int J Epidemiol **23**(2): 277-284.	Data overlapping Hammar 1994.
	Bosma, H., M. G. Marmot, H. Hemingway, A. C. Nicholson, E. Brunner and S. A. Stansfeld (1997). "Low job control and risk of coronary heart disease in Whitehall II (prospective cohort) study." Bmj **314**(7080): 558-565	Data overlapping Kuper 2002 (Whitehall II (1985, ~10,000 civil servants, ~250 IHD diagnoses).
	Brunner, E. J., M. Kivimäki, J. Siegrist, T. Theorell, R. Luukkonen, H. Riihimäki, J. Vahtera, J. Kirjonen and P. Leino-Arjas (2004). "Is the effect of work stress on cardiovascular mortality confounded by socioeconomic factors in the Valmet study?" J Epidemiol Community Health **58**(12): 1019-1020.	Overlapping more comprehensive report of the same data, Kivimaki 2004.
	Kivimäki, M., J. Head, J. E. Ferrie, E. Brunner, M. G. Marmot, J. Vahtera and M. J. Shipley (2006). "Why is evidence on job strain and coronary heart disease mixed? An illustration of measurement challenges in the Whitehall II study." Psychosom Med **68**(3): 398-401.	Data overlapping Kuper 2003.
	Kivimäki, M., S. T. Nyberg, G. D. Batty, M. J. Shipley, J. E. Ferrie, M. Virtanen, M. G. Marmot, J. Vahtera, A. Singh-Manoux and M. Hamer (2011). "Does adding information on job strain improve risk prediction for coronary heart disease beyond the standard Framingham risk score? The Whitehall II study." Int J Epidemiol **40**(6): 1577-1584.	Data overlapping Virtanen 2010.
	Clays, E., A. Casini, K. Van Herck, D. De Bacquer, F. Kittel, G. De Backer and A. Holtermann (2016). "Do psychosocial job resources buffer the relation between physical work demands and coronary heart disease? A prospective study among men." Int Arch Occup Environ Health **89**(8): 1299-1307.	Data overlapping DeBacker 2005.
	Ferrario MM, Veronesi G, Gianfagna F, Roncaioli M, Grassi G, Cesana G. Exploring the combined effect of job strain with levels of occupational and sport physical activity on cardiovascular disease incidence: The MONICA Brianza-PAMELA follow-up study. European Journal of Preventive Cardiology. 2017;24(2 Supplement 1):27.	Data overlapping Ferrario 2019 (same exposure and outcome).
	Magnusson Hanson, L. L., N. H. Rod, J. Vahtera, M. Virtanen, J. Ferrie, M. Shipley, M. Kivimäki and H. Westerlund (2020). "Job insecurity and risk of coronary heart disease: Mediation analyses of health behaviors, sleep problems, physiological and psychological factors." Psychoneuroendocrinology **118**: 104706.	Same study population as Ferrie 2012 with same risk estimates (besides mediation analyses).
**5. Other issues**		**Specific reasons for ineligibility**
	Aboa-Éboulé, C., C. Brisson, E. Maunsell, R. Bourbonnais, M. Vézina, A. Milot and G. R. Dagenais (2011). "Effort-reward imbalance at work and recurrent coronary heart disease events: a 4-year prospective study of post-myocardial infarction patients." Psychosom Med **73**(6): 436-447.	Analyses of recurrent AMI in patients with earlier hospitalisation for AMI.
	Kivimäki, M., S. T. Nyberg, G. D. Batty, M. J. Shipley, J. E. Ferrie, M. Virtanen, M. G. Marmot, J. Vahtera, A. Singh-Manoux and M. Hamer (2011). "Does adding information on job strain improve risk prediction for coronary heart	Overlapping Kuper 2003. Addresses the question whether adding job strain to the Framingham score (hypertension, lipids, smoking, diabetes) improves prediction of CHD.

	disease beyond the standard Framingham risk score? The Whitehall II study." Int J Epidemiol **40**(6): 1577-1584.	

V Studies by type of exposure

Number of studies and risk estimates by type of exposure type and selected study characteristics. Twenty-five follow-up studies and one case-control study 1994-2003 addressing risk of ischemic heart disease (IHD) according to the demands-control-support job stress model.

	All		Myocardial infarction		IHD unspecified		IHD mortality	
Exposure	N studies	N risk estimates	N studies	N risk estimates	N studies	N risk estimates	N studies	N risk estimates
Demands	16	35	7	14	6	16	3	5
Control	20	38	9	17	8	17	3	4
Social support	8	14	3	4	4	8	1	2
Job strain	21	32	11	14	8	15	2	3
Isostrain	2	3	0	0	1	1	1	2
All	26	122	13	49	10	57	3	16
Repeated exposure assessments	3	14	1	1	1	3	1	10
Exposure response data	14	73	6	29	6	38	2	6
Cumulative exposure assessment	2	11	1	1	0	0	1	10
Analysis of interaction between demands and control	3	15	2	12	0	0	1	3

VI Covariates

Table 4. Covariates in follow-up studies reported in 26 publications 1994-2023 addressing risk of ischemic heart disease and the demands-control-support job stress model.

		All N=26	Myocardial infarction N=13	IHD unspecified N=10	IHD mortality N=3
Socioeconomic variables					
	Education	21	10	9	2
	Income	11	9	1	1
	Occupational class or grade	18	9	6	3
	Marital status	10	4	5	1
	By design[1]	5	2	3	0
Potential confounders					
	Ethnicity or race	9	5	3	1
	Family history of cardiovascular disease	5	2	2	1
	Personality	2	1	1	0
	Comorbidity	6	3	3	0
	Childhood adversity	2	0	1	1
	Social network	8	3	4	1
	Residence	5	2	3	0
	Calendar year	6	1	3	2
	Other work-related exposures	9	4	3	2
Potential modifiers and/or confounders					
	Physiological risk factors (hypertension, hyperlipidaemia, diabetes)	17	9	7	1
	Behavioural risk factors (smoking, alcohol, physical activity)	20	11	8	1
	Distress, depression	5	3	1	1

VII Funnel plot job strain and ischemic heart disease

Funnel plot of risk of associations between high job strain and ischemic heart disease in eligible published studies (n=26). Dots represent actual RREs, while circles represent possible RREs which should exist if no publication bias was present.

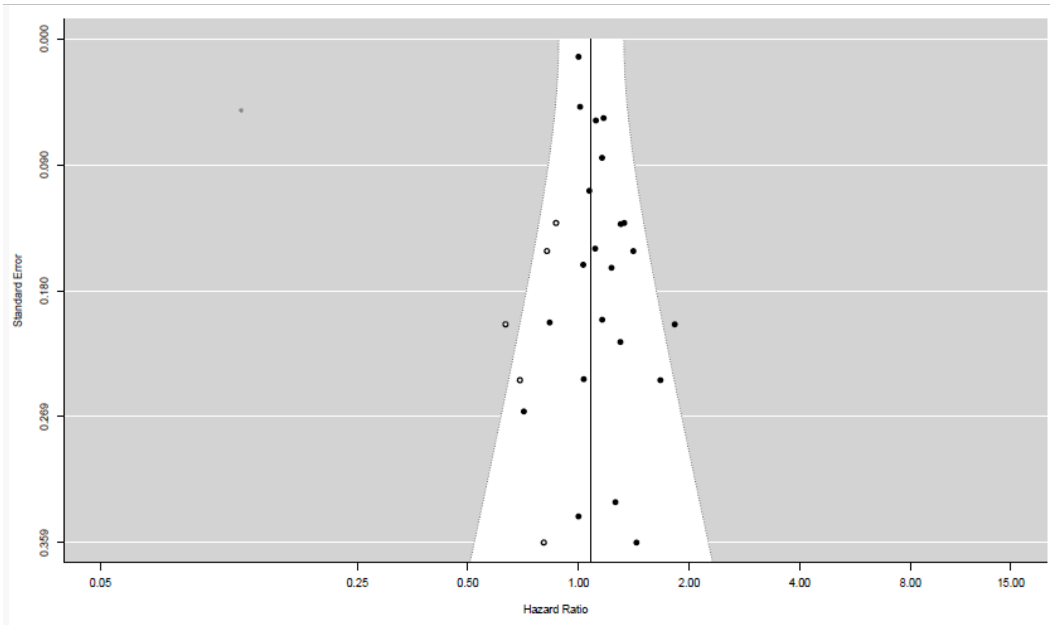

VIII Forest plot of high isostrain and ischemic heart disease

Forest plot of relative risk estimates (RRE)[1] of ischemic heart disease by high versus low isostrain in eligible published studies (N=2 studies with 3 risk estimates). Abbreviations are defined in Supplemental Material X.

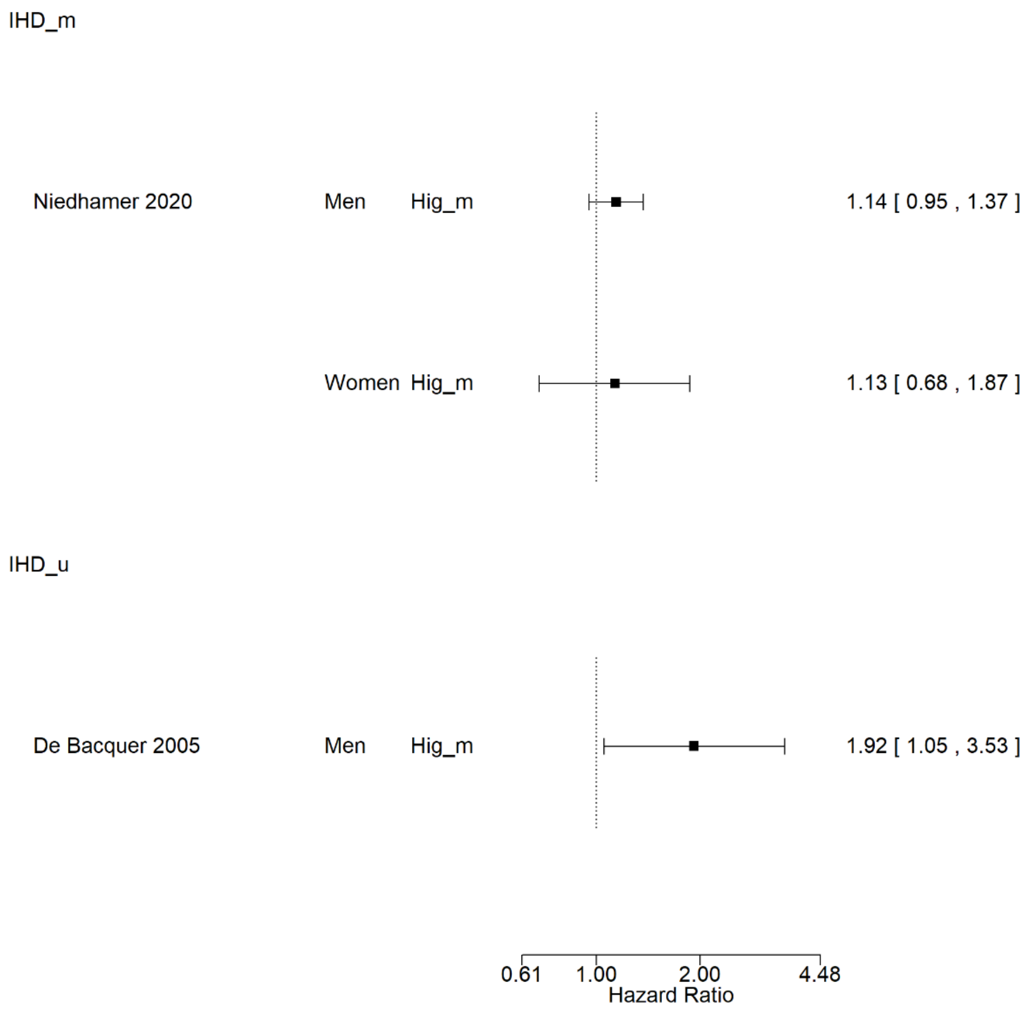

[1] 'RRE' (relative risk estimate) refers to approximative relative risks in studies with rare outcomes (true relative risks, hazard ratios and odds ratios). The meta-analytic weighted estimate across studies is not provided given strong heterogeneity across studies.

IX Forest plot of high job demands and ischemic heart disease

Forest plot of relative risk estimates (RRE)[1] of ischemic heart disease by higher versus lowest level of job demands in eligible published studies (N=16 studies with 35 risk estimates). Abbreviations are defined in Supplemental Material X.

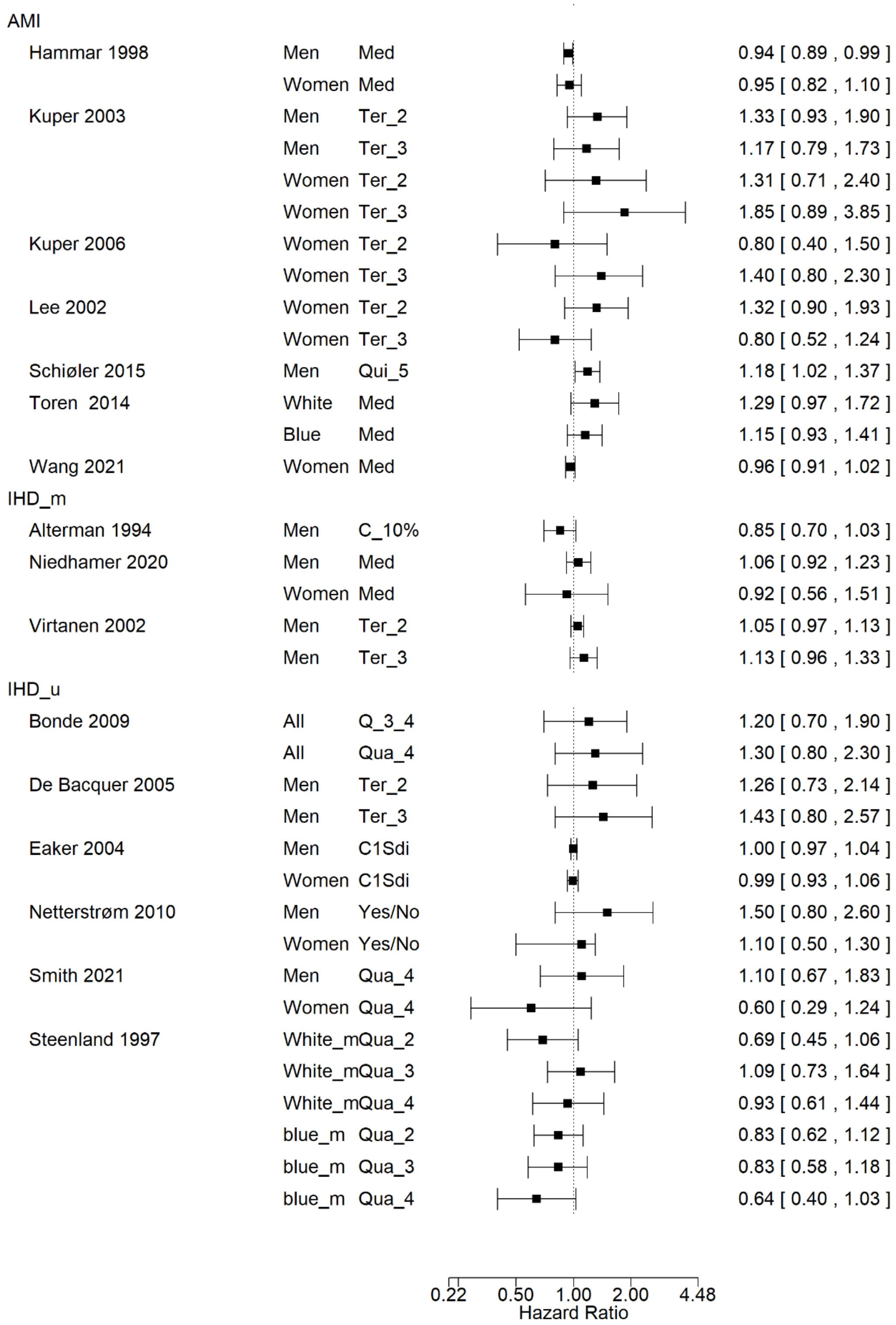

[1] 'RRE' (relative risk estimate) refers to approximative relative risks in studies with rare outcomes (true relative risks, hazard ratios and odds ratios). The meta-analytic weighted estimate across studies is not provided given strong heterogeneity across studies.

X Forest plot of low job control and ischemic heart disease

Forest plot of relative risk estimates (RRE)[1] of ischemic heart disease by lower versus highest level of job control in eligible published studies (N=20 studies with 38 risk estimates). Abbreviations are defined in Supplemental Material X.

AMI			
Hammar 1998	Men	Med	1.19 [1.13 , 1.25]
	Women	Med	1.44 [1.25 , 1.65]
Huisman 2008	All	Med	1.42 [0.97 , 2.08]
Kuper 2003	Men	Ter_2	1.32 [0.97 , 1.79]
	Men	Ter_3	1.01 [0.70 , 1.45]
	Women	Ter_2	0.70 [0.30 , 1.64]
	Women	Ter_3	0.92 [0.45 , 0.89]
Kuper 2006	Women	Ter_2	0.80 [0.50 , 1.40]
	Women	Ter_3	0.70 [0.40 , 1.20]
Lee 2002	Women	Ter_2	0.81 [0.54 , 1.22]
	Women	Ter_3	0.97 [0.65 , 1.45]
Schiøler 2015	Men	Qui_5	1.13 [0.98 , 1.31]
Toren 2014	White	Med	1.06 [0.84 , 1.33]
	Blue	Med	1.23 [0.98 , 1.56]
Vaananen 2008	All	C1SDd	1.04 [0.93 , 1.16]
	All	C1SDd	1.12 [0.98 , 1.28]
Wang 2021	Women	Med	1.03 [0.97 , 1.09]
IHD_m			
Alterman 1994	Men	C_20%	1.32 [0.95 , 1.82]
Niedhamer 2020	Men	Med	1.14 [0.98 , 1.46]
	Women	Med	1.26 [0.67 , 2.39]
Virtanen 2002	Men	Ter_3	1.11 [1.04 , 1.19]
IHD_u			
Bonde 2009	All	Q_3_4	1.40 [0.80 , 2.40]
	All	Qua_4	2.00 [1.10 , 3.60]
De Bacquer 2005	Men	Ter_2	0.73 [0.42 , 1.26]
	Men	Ter_3	0.83 [0.48 , 1.43]
Eaker 2004	Men	C1SDd	1.00 [0.94 , 1.05]
	Women	C1SDd	0.54 [0.35 , 0.83]
Hemmingsson 2006	Men	Qua_2	0.82 [0.68 , 0.99]
	Men	Qua_3	0.77 [0.64 , 0.93]
	Men	Qua_4	0.99 [0.82 , 1.19]
Hibbard 1993	Men	C1SCi	1.00 [0.91 , 1.11]
	Women	C1SCi	0.91 [0.83 , 1.11]
Netterstrøm 2010	Men	Yes/No	1.11 [0.77 , 2.00]
	Women	Yes/No	0.83 [0.32 , 2.00]
Smith 2021	Men	Qua_4	0.72 [0.42 , 1.24]
	Women	Qua_4	0.78 [0.42 , 1.43]
Steenland 1997	White	Qua_4	1.35 [0.79 , 2.33]
	Blue	Qua_4	1.45 [0.98 , 2.17]

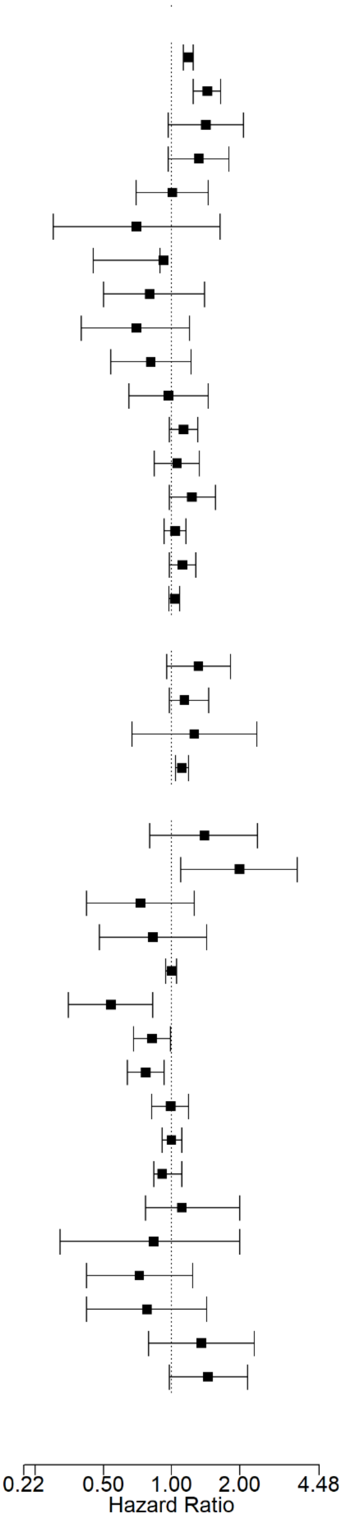

[1] 'RRE' (relative risk estimate) refers to approximative relative risks in studies with rare outcomes (true relative risks, hazard ratios and odds ratios). The meta-analytic weighted estimate across studies is not provided given strong heterogeneity across studies.

XI Forest plot of low workplace social support and ischemic heart disease.

Forest plot of relative risk estimates (RRE)[1] of ischemic heart disease by lower versus highest level of workplace social support in eligible published studies (N=8 studies with 14 risk estimates). Abbreviations are defined in Supplemental Material X.

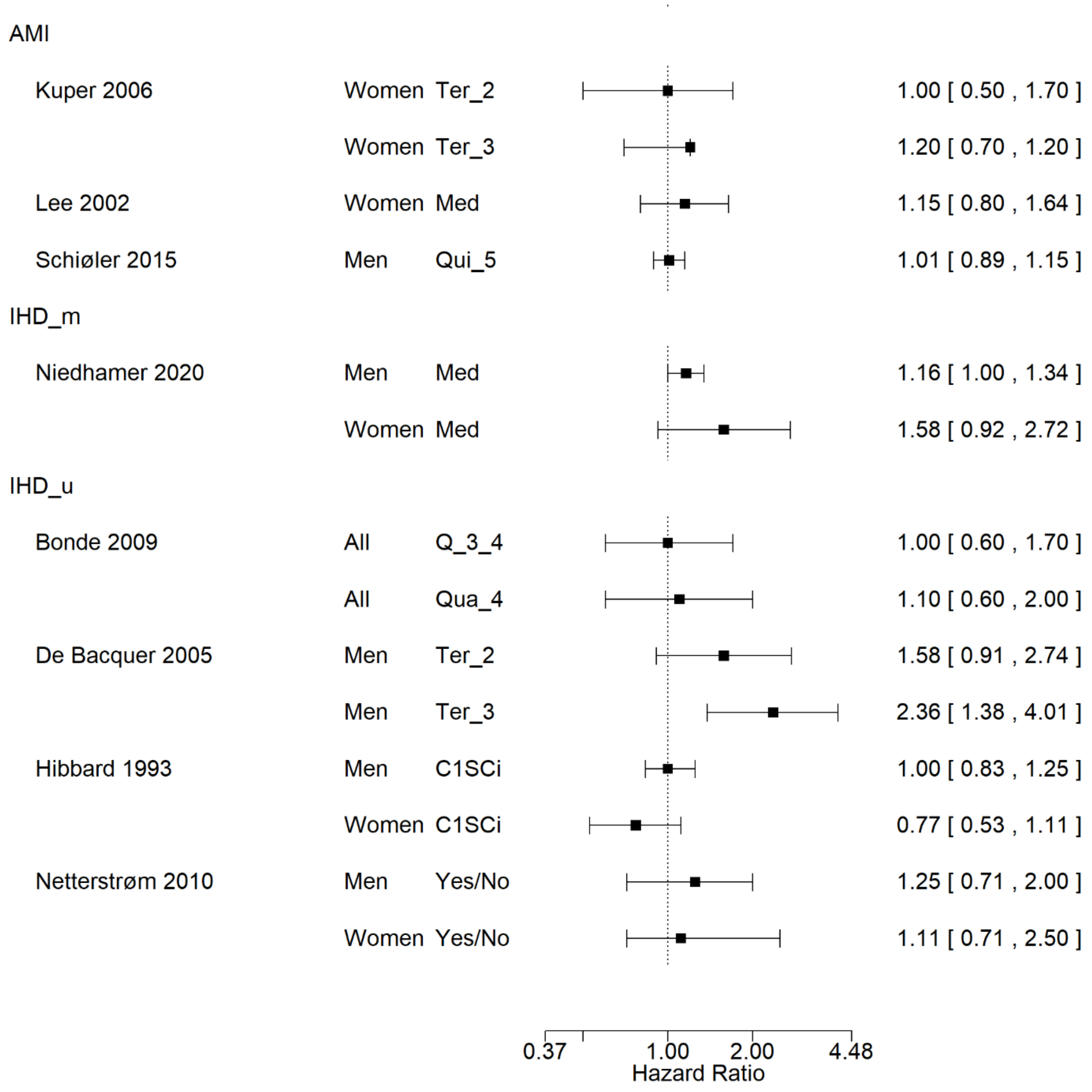

[1] 'RRE' (relative risk estimate) refers to approximative relative risks in studies with rare outcomes (true relative risks, hazard ratios and odds ratios). The meta-analytic weighted estimate across studies is not provided given strong heterogeneity across studies.

XII List of abbreviations in tables and forest plots

IHD	Ischemic heart disease (ICD-10: I20-I25, ICD-8-9: 410)
AMI	Myocardial infarction (ICD-10: I21, ICD-8-9: 410-414)
CI	Confidence interval
HR	Hazard ratio
OR	Odds ratio
NR	Not reported
NA	Not applicable
JEM survey	Job exposure matrix based upon independent occupational surveys of self-reported workplace psychosocial exposures. Job specific item scores are assigned on the basis of the job title at baseline unless otherwise specified.
JEM expert	Job exposure matrix based upon expert assessment, observations, and measurements.

Job strain, quadrant, median, high vs low:

Combination of high demands and low control defined by median scale score in which high strain is compared to low strain (low demands and high control).

Job strain, quadrant, median, high vs all:

Combination of high demands and low control defined by median scale sum score in which high strain is compared with all other demand/control combinations.

Job strain, quadrant, tertiles, high vs low:

Combination of high demands and low control defined by tertile of item sum score in which high strain (highest tertile of demand and low/intermediate control) is compared to low strain (low demands and high control)

Job strain ratio method:

Ratio of demand and inversed control item scores dichotomised >=1 (highest strain) and <1 (lowest strain)

Isostrain, quadrant, median split:

Job strain combined with low social support at work (median split), high isostrain vs low isostrain.

Abbreviations in forest plots

Outcomes

AMI	Myocardial infarction
IHD_u	Ischemic heart disease unspecified
IHD_m	Ischemic heart disease mortality

Strata within studies

White_c	White collar
Blue_c	Blue collar
White_m	Male white collar
Blue_m	Male blue collar
White_w	Female white collar
Blue_w	Female blue collar

Exposure contrast

Med_R	High versus low job strain defined by median scale score, quadrant method, median split, strain versus relaxed
Med_0	High versus low job strain defined by median scale score, quadrant method, median split strain versus all others
Ter_2	Second versus lowest tertile of scale scores
Ter_3	Third versus lowest tertile of scale scores
Qua_2	Second versus lowest quartile of scale scores
Qua_3	Third versus lowest quartile of scale scores
Qua_4	Fourth versus lowest quartile of scale scores
Q_3_4	Third or fourth versus lowest quartile of scale scores
Qui_5	Fifth versus lowest quintile of scale scores
Med	Scale score above median versus <= median
Hig_m	High isostrain (high strain and low support, median split)
